# Infrared radiation from cage bedding moderates rat inflammatory and autoimmune responses in collagen-induced arthritis

**DOI:** 10.1038/s41598-021-81999-7

**Published:** 2021-02-03

**Authors:** Jasmina Djuretić, Mirjana Dimitrijević, Marija Stojanović, Jelena Kotur Stevuljević, Michael R. Hamblin, Ana Micov, Radica Stepanović-Petrović, Gordana Leposavić

**Affiliations:** 1 Department of Pathobiology, Faculty of Pharmacy, University of Belgrade, Vojvode Stepe 450, Belgrade, Serbia; 2 Department of Immunology, Institute for Biological Research “Siniša Stanković”-National Institute of Republic Serbia, University of Belgrade, Bulevar despota Stefana 142, Belgrade, Serbia; 3 Department of Biochemistry, Faculty of Pharmacy, University of Belgrade, Vojvode Stepe 450, Belgrade, Serbia; 4 Laser Research Centre, Faculty of Health Science, University of Johannesburg, Doornfontein, 2028 South Africa; 5 Department of Pharmacology, Faculty of Pharmacy, University of Belgrade, Vojvode Stepe 450, Belgrade, Serbia

**Keywords:** Pain, Rheumatoid arthritis

## Abstract

The development of collagen type II (CII)-induced arthritis (CIA), a model of rheumatoid arthritis, in rats housed in cages with bedding composed of Celliant fibres containing ceramic particles, which absorb body heat and re-emit the energy back to the body in the form of infrared radiation (+IRF rats), and those housed in cages with standard wooden shaving bedding (−IRF control rats) was examined. The appearance of the first signs of CIA was postponed, while the disease was milder (judging by the arthritic score, paw volume, and burrowing behaviour) in +IRF compared with −IRF rats. This correlated with a lower magnitude of serum anti-CII IgG antibody levels in +IRF rats, and lower production level of IL-17, the Th17 signature cytokine, in cultures of their paws. This could be partly ascribed to impaired migration of antigen-loaded CD11b + dendritic cells and their positioning within lymph nodes in +IRF rats reflecting diminished lymph node expression of CCL19 /CCL21. Additionally, as confirmed in rats with carrageenan-induced paw inflammation (CIPI), the infrared radiation from Celliant fibres, independently from immunomodulatory effects, exerted anti-inflammatory effects (judging by a shift in pro-inflammatory mediator to anti-inflammatory/immunoregulatory mediator ratio towards the latter in paw cultures) and ameliorated burrowing behaviour in CIA rats.

## Introduction

As a result of substantial advances in polymer chemistry, relatively recently, infrared radiation-emitting (IR) fibres (filaments) have been developed. These fibres have a porous core-sheath and groove structure allowing various optically active ceramic micron-sized particles to be incorporated into each fibre^1,2^. One type of these fibres is Celliant (Hologenix, Santa Monica, CA, USA), a polyethylene terephthalate fibre that incorporates micron-sized optically active ceramic particles exhibiting the property of temperature-dependent infrared emission. These fibres have been woven to get high-performance functional textiles^1,2^. When such functional textiles are used as garments, bandages, or bed linen, the heat energy generated by the human body can be transferred by radiation, conduction, convection to the ceramic particles^1–4^. These ceramic particles act as black-body absorbers and re-emit the absorbed energy as infrared radiation back to the body^5^. According to the classification of the International Commission on Illumination (CIE) and the classification provided in ISO 20473 standard (ISO 20473), infrared radiation has three broad categories: near infrared (0.7–1.4 μm and 0.78–3 μm according to CIE and ISO 20473, respectively), mid-infrared (0.4–3 μm and 3–50 μm according to CIE and ISO 20473, respectively) and far infrared (3–100 μm and 50–1000 μm according to CIE and ISO 20473, respectively). Considering the aforementioned data, IR fabrics are suggested to recycle the body’s natural energy^5^. At physiological skin temperature, much of the body’s emissive radiative power is centered between 7 and 14 μm^6,7^. Generally, infrared radiation penetrates deeply through the layers of the skin to reach the muscles and bones^8^. This radiation moderates inflammation^9,10^ and pain^4^. Additionally, it promotes tissue regeneration and wound healing by improving circulation^11,12^ and/or acting directly on cells to improve mitochondrial metabolism^13^, and thereby a number of cellular functions including energy generation, calcium signaling, and cell growth^14^. Given that infrared radiation is non-invasive and painless, it could be a broadly applicable therapeutic option for moderating inflammation and pain ^2,8^. However, when this radiation is delivered from standard electrically-powered sources (such as IR heat lamps and IR saunas), it could cause a prolonged erythemal response due to excessive heating of the skin^2^. Additionally, when the radiation is delivered from these sources, it is difficult to delineate effects related to the increase in the core body temperature (hyperthermia) from the direct biochemical effects on living cells^2^. It is noteworthy that the infrared radiation that does not produce any detectable skin heating effects, such as the infrared radiation emitted by the ceramic particles enriched IR fabrics^2^, can also produce biological effects^15,16^. Indeed, IR fabrics that mainly rely on the energy emitted from the body have been found to reduce inflammation and pain^15^, so their therapeutic use may be considered. In this context, it should be added that IR fabrics, may be worn for extended periods in the form of clothing or bandages or used as bed linen to attain health benefits^2^. Rheumatoid arthritis (RA) is a chronic inflammatory autoimmune disease primarily affecting the lining of the synovial joints, and causing progressive disability, premature death, and high socioeconomic burdens^17,18^. The clinical manifestations encompass symmetrical joint involvement including arthralgia (joint pain), swelling, redness, and even a limited range of motion^17,18^. Many immune and other cell types and their cytokines play roles in the development of RA^17,18^. The synovial compartment is infiltrated with adaptive immune cells, including both T cells and B cells, and innate immune cells (monocytes and macrophages), which interact between themselves and with fibroblast-like synoviocytes to produce inflammatory mediators^17^. Effector Th17 cells acting together with arthritogenic autoantibodies are suggested to be the major driver of non-resolving joint tissue damage, and therefore prolonged inflammation in RA^17,19^. Monocytes/macrophages massively infiltrating the synovial membranes in RA^20,21^, are shown to be central to the joint inflammation^22^. The imbalance between monocyte/macrophages with pro-inflammatory secretory profile and monocyte/macrophages with anti-inflammatory/immunoregulatory secretory profile is suggested to be particularly important for RA development, as a shift towards the former contributes to osteoclastogenesis (i.e. production of osteoclasts, the cells specialized for bone resorption), and thereby to bone loss that ultimately leads to the destruction of the subchondral bone and the degeneration of the overlying articular cartilage ^22,23^. On the other hand, a shift towards monocyte/macrophages with anti-inflammatory/immunoregulatory secretory profile has been suggested to contribute to the regression of joint injury and inflammation in RA^24^.

While there is currently no long-term cure for RA, the treatment strategy aims to alleviate arthralgia and rapidly achieve a lowering of the disease activity state^17^. The introduction of novel disease-modifying anti-rheumatic drugs (DMARDs) has dramatically improved the prognosis of RA patients, but a significant proportion of these patients fail to report long-term relief of arthralgia, reflecting the incomplete disease control^17^. In this context, significant efforts have been made to show that is important to put inflammation under control in the early phases of RA development^25^. Additionally, even if treatment with DMARDs does reduce arthralgia, a proportion of RA patients is still dissatisfied with its management and continue to rate the pain relief as one of their top requirements for improved health and quality of life^17^. Considering various side effects (including gastrointestinal disorders, immunosuppression, and humoral disturbances) of analgesics, which are most commonly used in RA, i.e. nonsteroidal anti-inflammatory drugs and corticosteroids, research into new options to control arthritis and arthralgia in RA is of great importance.

The most widely used RA model is rodent collagen type II (CII)-induced arthritis (CIA). This model has gained acceptance since it is reproducible, well defined, and particularly because it has proven useful for the development of new therapies for RA^26^. It has also been recommended to use rats for studying the anti-arthritic effects of various agents, as rats are less variable than mice, their joints are bigger and the inflammatory changes are more reproducible^26^.

The present study was primarily undertaken to examine the effects of exposure of rats to IR fibres used as cage bedding (mimicking exposure to IR fabrics used as bed linen) on the development of autoimmune inflammation of joints in the CIA model. The study included female Dark Agouti (DA) rats as compared with male rats, they exhibit a substantially higher incidence of CIA and more severe disease^27–29^. In these rats, joint inflammation and burrowing behaviour were examined. We decided to evaluate burrowing behaviour as it was suggested to be a reliable tool to measure outcomes similar to those measured in the clinical trial evaluating effects of analgesics in chronic pain conditions (viz. spontaneous pain and overall patient healthy status), as it is RA^30^. Additionally, to elucidate the putative mechanisms standing behind the effects of infrared radiation on the development of the inflammation of paw joints in CIA rats, the indicators of ongoing humoral (the serum levels of anti-CII-specific antibodies) and cellular (the production levels of IL-17, Th17 signature cytokine, in inflamed paw cultures from CIA rats) immune responses, and inflammatory response (the production levels of the key pro-inflammatory cytokines, i.e.TNF-α, IL-1β and PGE2 and NO, and anti-inflammatory/immunoregulatory mediators IL-10 and TGF-β in RA, in inflamed paw cultures)^31–36^ were examined. The main source of these mediators in RA are suggested to be activated macrophages ^31–36^ Of note, macrophage activation is shown to be a dynamic process; the same cells may initially take part in proinflammatory and cytotoxic reactions and later participate in the resolution of inflammation and wound healing, so in an inflammatory microenvironment, they are “blend” together critically shaping the outcome of the inflammation^37^. Given that the primary results showed that the exposure to IR fibres as cage bedding moderated development of both humoral and cellular immune responses, and that joint tissue-specific antibodies in RA/CIA may cause arthralgia in the absence of overt inflammation through direct action on sensory neurons^38^, we extended our research to study the effects of exposure to IR fibres as cage bedding to the development of carrageenan-induced paw inflammation (CIPI), a model commonly used to assess the production of inflammatory mediators at sites of inflammation, the anti-inflammatory properties of agents such as nonsteroidal anti-inflammatory drugs, and the efficacy of putative analgesic compounds to reverse cutaneous hypersensitivity^39^. Given that rats from cages with bedding from IR fibres developed paw inflammation of lower magnitude compared with those from cages with standard wood shaving bedding, the effects of rat exposure to IR fibres in a treatment paradigm were examined, as well.

## Materials and methods

### Animals

In the present study four-month-old female Dark Agouti (DA) rats from a breeding colony in the Immunology Research Centre “Branislav Janković” (Belgrade, Serbia) were used. Rats were maintained in a fully controlled animal facility with a constant temperature (21–23 °C) and humidity (30–50%), a 12 h/12 h light/dark cycle, and they were provided ad libitum access to water and standard pelleted food. All experiments were performed in accordance with the Directive 2010/63/EU of the European Parliament and of the Council on the Protection of Animals used for Scientific Purposes (revised Directive 86/609/EEC), and were approved by Laboratory Animal Ethical Committee of University of Belgrade—Faculty of Pharmacy (Etička Komisija za ogledne životinje, Univerzitet u Beogradu –Farmaceutski fakultet).

### Induction and clinical evaluation of CIA

Rats were immunised intradermally at the base of the tail with 300 μg of bovine CII Sigma-Aldrich Chemie GmbH, Taufkirchen, Germany) emulsified in incomplete Freund's adjuvant (IFA), as previously described^27,28^. The immunisation emulsion was prepared by mixing equal volumes of CII solution (2 mg/mL) in 0.1 M acetic acid and IFA. For immunisation 300 µL of this emulsion was injected per rat. Before the immunisation, animals were anesthetized with an intraperitoneal injection of anesthetic cocktail [50 mg/kg/body weight (BW) of ketamine/5 mg/kg BW xylazine; Ketamidor, Richter Pharma AG, Wels, Austria; Xylased, Bioveta, Ivanovice na Hané, Czech Republic]. Clinical signs of arthritis were evaluated daily from the 7th day post immunisation (d.p.i.) until the 22nd d.p.i., when the clinical severity of the disease reaches the maximum ^27,28^. The severity of CIA was graded according to an arbitrary scale taking into account joint edema and erythema of the hind and front paws (one point for each inflamed metacarpophalangeal/metatarsophalangeal or interphalangeal joint, and five points for the inflamed ankle)^40^ by two experienced researchers (MD and MS), independently. Thus, each paw could receive the maximum score of 15 points giving the highest arthritic score of 60. Additionally, paw volumes were measured before the immunisation (basal) and on the 22nd d.p.i. Given that pathological changes predominantly occurred in the hind paws of rats, volumes of both hind paws were measured. Of note, these measurements and all subsequent analyses were conducted by two investigators blind to the rat cage bedding.

### Induction and clinical evaluation of CIPI

Paw inflammation was induced by intraplantar injection of 1.5 mg carrageenan (λ-carrageenan, Sigma Aldrich, St.Louis, MO, USA) in 150 μL saline or 150 μL of saline (controls) into the right hind paw starting from the midline near the heel and continuing toward the base of the second or third toe as previously described^39^. The volumes of their right hind paws were measured before injection of carrageenan or saline (basal) and four times afterward at one hour intervals. The temperature of the right hind paws was measured using a digital touch-free thermometer (Microlife AG, Widnau, Switzerland) at the same time points. Of note, these measurements and all subsequent analyses were conducted by two investigators blind to the rat cage bedding.

### Experimental design

For the induction of CIA rats were randomly assigned into two groups (10 rats per group). Five days before immunisation one group of rats was housed in cages with bedding composed of IR Celliant fibres (+IRF rats), while the other group of rats was housed in cages with standard wood shaving bedding (−IRF rats). Celliant is produced using a total of 13 naturally occurring, thermoreactive minerals. These include: titanium dioxide, a photocatalyst that effectively absorbs light, silicon dioxide, which absorbs and reflects energy, and aluminium oxide, which can help increase energy reflectivity. All 13 minerals are ground up into an ultra-fine powder and then mixed with polyethylene terephthalate to create the ‘Celliant master batch’. Finally, a liquid polyester resin is added to the master batch and synthesized into a stable fibre. The cage-bedding was constructed from 16 g Celliant (200 g/m^2^) of 42 mm fibres (thickness less than 10 μ, ceramic loading 1–1.25% by weight) and standard wood shavings (1:10 weight/weight) (Fig. S1). According to the manufacturer, the ceramics absorb the body’s heat, transforming it into full-spectrum infrared energy (https://celliant.com/how-it-works) with a broad-peak cantered at a wavelength of 9.3 µm, whereas the power density depends on the proximity of the heat source (living body), but is approximately 0.25 mW/cm^2^, so for each hour, an energy density of about 1 J/cm^2^ is delivered. The standard wood shaving and IR Celliant beddings were changed every second day. The burrowing behaviour test was performed on the 21st d.p.i. Following the test animals were anesthetized with an intraperitoneal injection of ketamine/xylazine anesthetizing cocktail (80 mg/kg BW ketamine/8 mg/kg BW xylazine) and blood was taken by cardiac puncture. Additionally, from euthanized rats were removed lymph nodes, spleens, and hind paws for further analysis.

To examine the effects of rat exposure to IR fibres on CIPI, two sets of experiments (each consisting of two separate experiments) were performed. In the first set of experiments, rats were randomly assigned into three groups (6 rats per group). Two groups were administered with carrageenan, whereas one group (6 rats) was administered with saline (SAL rats). Five days before administration of rat hind paws with carrageenan one group of animals was transferred to cages with Celliant bedding (+IRF rats), while the other groups remained in cages with standard wooden shaving bedding (−IRF rats). In the second set of experiments, rats were randomly assigned into three groups (6 rats per group). Following the administration of right hind paws with carrageenan one group of rats was transferred to cages with Celliant bedding (+IRF rats), one group of rats was administered with 5 mg/kg Diclofenac (Diklofen, Galenika AD, Belgrade, Serbia) per os (DIC rats), whereas one group of rats remained in cages with standard bedding (−IRF rats). The dose of Diklofen was chosen to correspond to that used in humans^41^.

In one experiment from each set of experiments 240 min following the carrageenan/saline administration burrowing test (lasting for 120 min) was started. At the end of this test, rats were euthanized by an intraperitoneal injection of ketamine/xylazine anesthetizing cocktail (80 mg/kg BW ketamine/8 mg/kg BW xylazine), and their right paws were retrieved for culturing. In the second experiment from each set of experiments, right paw volume and mechanical hyperalgesia were examined at one hour intervals over 240 min from the carrageenan/saline administration.

### Paw volume measurement

Paw volume was measured using a plethysmometer (Ugo Basile, Gemonio, Lombardy, Italy), as described previously^42^. The average of two consecutive volume measurements for each rat was used for further calculations. The results are expressed as the difference (dV) between post-immunisation or CIPI induction and the basal paw volume according to the following formula^42^:

$${\text{dV }} = {\text{ volume of the inflamed paw }}\left( {{\text{mL}}} \right) \, - {\text{ basal volume of the same paw }}\left( {{\text{mL}}} \right)^{{}}$$

Next, the effect of treatment (Celliant fibres or Diklofen) on dV was calculated according to the following formula

$${\text{dV reduction }}\left( \% \right) \, = \frac{{{\text{referent group}}*{\text{ average dV }} - {\text{ dV of each rat from experimental group}}**}}{{\text{referent group average dV}}} \times 100$$

* CIA/CIPI rats from cages with standard bedding without any treatment; ** rats exposed to Celliant fibres or administered with Diklofen/saline^42^.

### Burrowing training and burrowing test

To assess influence of Celliant bedding on spontaneous joint pain, burrowing test, the test developed with the goal of enhancing the translational potential of preclinical findings in pain research^43^ was used. This test examines burrowing behaviour, an ancient adaptive behaviour conserved across many rodent species, one in which various laboratory strains of mice and rats spontaneously engage, and, more important, one which is depressed in both mice and rats experiencing inflammatory and neuropathic pain^44,45^. It is noteworthy, that this behaviour is considered to represent a correlate of so-called “activities of daily living” in humans—tasks that are essential to satisfactory quality of life and are often impeded by pain^46^. For burrowing training and experiments, long plastic tubes (32 cm in length and 10 cm in diameter), with the open-end elevated 6 cm from the cage bottom, were filled with 2500 g of gravel (2–6 mm diameter particles). The training was performed four days before housing in cages with Celliant bedding, and both training and burrowing behaviour tests were performed during the dark phase of the daily cycle starting at 6 pm. The training was conducted in social facilitation and individual training formats. For social facilitation, the rats from one cage were placed in a cage-burrow setup for 120 min. To estimate burrowing behaviour, the weight of the burrowed gravel was calculated (weight of gravel left in the tube after completion of burrowing training or test was subtracted from the initial weight of gravel in the tube). Individual training was performed so that a single rat was placed in a cage-burrow setup for 120 min per day for three consecutive days and the average amount of burrowed gravel was determined as explained above. Rats burrowed less than 500 g (four out of 56) were classified as poor burrowers and they were excluded from further experiments as previously suggested^46^. The test was performed before immunisation/inflammation induction (to assess basal burrowing activity) and at a certain point following the immunisation/inflammation induction.

Following the test burrowing activity (BA) of each rat was calculated according to the following formula:

$${\text{BA }}\left( \% \right) \, = \frac{{{\text{BA after immunisation}}/{\text{inflammation induction }}\left( {\text{g}} \right)}}{{\text{Basal BA}}} \times 100$$

and then increase in burrowing activity was calculated according to the following formula:

$${\text{BA increase }}\left( \% \right) \, = \frac{{{\text{BA of each rat from treated group }}* - {\text{ BA of referent group}}** \, \left( {{\text{average}}} \right)}}{{{\text{BA of referent group }}\left( {{\text{average}}} \right)}} \times 100$$

* rats exposed to Celliant fibres or rats administered with Diklofen; **rats from cages with standard bedding.

### Electronic Von Frey test

The mechanical hyperalgesia following carrageenan injection was assessed by measuring paw withdrawal thresholds (P, expressed in g) using an electronic Von Frey anesthesiometer (IITC Life Science, Woodland Hills, CA, United States) as described previously^47,48^. The rats were placed in transparent boxes on the top of a metal grid and allowed to acclimatize for 30 min before testing. A plastic, semi-flexible filament coupled with a force transducer was used to deliver the mechanical stimulus. The tip of the filament was applied perpendicularly to the plantar surface of the right hind paw and the pressure was gradually increased until the rat withdrew its paw (that pressure was recorded automatically on a digital screen). Basal Ps were measured before inflammation induction. Basal and post-induction Ps were measured on the right hind paw. The results are expressed as the difference (dP) between basal and post-induction Ps according to the following formula^47,48^:

$${\text{dP }} = {\text{ P before inflammation induction }}\left( {\text{g}} \right) \, - {\text{ P after inflammation induction }}\left( {\text{g}} \right)$$

Treatment reducing dP was recognized as antihyperalgesic treatment. The percentage of the antihyperalgesic activity (AHA) was calculated as previously suggested^48^:

$${\text{AHA }}\left( \% \right) \, = \frac{{{\text{dP of referent group}}* \, \left( {{\text{average}}} \right) \, - {\text{ dP of each rat in treated group}}**}}{{{\text{dP of referent group }}\left( {{\text{average}}} \right)}} \times 100$$

* rats from cages with standard bedding; ** rats exposed to Celliant fibres or rats administered with Diklofen.

### Isolation of mononuclear cells

To obtain mononuclear single cell suspensions from lymph nodes (LNs) for the analysis of migratory capacity of innate immune cells in fluorescein isothiocyanate (FITC) painting test and spleens for in vitro analyses of adherence and phagocytic capacity of innate immune cells, LNs draining the site of FITC application (DLNs) and spleens were dissociated using a 70 µm nylon cell strainer (BD Biosciences, Erembodegem, Belgium) and obtained cells were collected in phosphate buffered saline (PBS) supplemented with 2% fetal calf serum (FCS, Gibco, Grand Island, NY, USA) and 0.01% NaN_3_ (Sigma-Aldrich Chemie GmbH) (FACS buffer), respectively. The single cell splenocyte suspensions were subjected to NH_4_Cl lysis to remove red blood cells and then washed in ice-cold FACS buffer. The mononuclear single cell suspensions from DLNs and spleen of each animal were enumerated using an improved Neubauer hemacytometer and trypan blue dye to exclude non-viable cells and adjusted to 1 × 10^7^ cells/mL.

### Flow cytometry analysis (FCA)

Briefly, for FCA single cell suspensions from DLNs and spleen were subjected to direct or indirect immunolabeling as previously described in details^49^. After immunolabeling 50,000 cell events per sample were acquired on a FACSCalibur flow cytometer (Becton–Dickinson, Mountain View, CA, USA). Data were analysed using FlowJo software version 7.8. (TreeStar Inc, Ashland, OR, USA). Dead cells and debris were excluded from the analyses by selective gating based on forward scatter (FSC) and side scatter (SSC).

### Innate immune cell functional assays

To assess the migration capacity of innate immune cells FITC painting test was used^50^. Briefly, rat left flank was shaved and painted with 300 μL of 5 mg/mL FITC dissolved in equal volumes of acetone and dibutylphthalate. After 24 h, ipsilateral (the side subjected to FITC painting) inguinal and axillary DLNs were extirpated and divided into two portions. From one portion single mononuclear cell suspensions were prepared as described above, whereas another portion was used to examine CCL19 and CCL21 expression using Reverse Transcription-Quantitative Polymerase Chain Reaction. Aliquots of 100 µL of DLN suspensions were processed for indirect immunolabeling with biotin-conjugated anti-CD11b and PerCP-conjugated streptavidin as primary and second step reagent, respectively. Following washing, cells were acquired for analysis using a FACSCalibur flow cytometer as described above. Results were expressed as % of FITC + cells within the CD11b + LN cells.

Innate immunity cells isolated from spleen were tested for cell adherence ability using a modification of previously described method^51^. Aliquots of 500 µL of splenocyte suspension (1 × 10^6^ cells/ml) were placed in an adherence column consisting of a 1 mL syringe packed with 50 mg of nylon fibres to a height of 1.25 cm. After 10 min, the effluent containing the non-adherent cells was drained by gravity. Initial cell suspensions and effluents were processed for immunolabeling with FITC-conjugated anti-CD11b (clone ED8; Serotec, Oxford, UK) antibody. The cells were incubated with saturating concentrations of the fluorochrome-labeled antibody for 30 min, washed with FACS buffer, and subsequently subjected for FCA. Results were expressed as

$${\text{adherence index }} = \frac{{\% {\text{ of total CD11b}} + {\text{ cells }} - \, \% {\text{ of non-adherent CD11b}} + {\text{ cells}}}}{{\% {\text{ of total CD11b}} + {\text{ cells}}}} \times 100\%$$

To assess the phagocytic capacity of innate immune cells a previously described method was used^49^. Briefly, 100 µl aliquots of splenocyte suspensions (1 × 10^6^ cells/ml) described, were incubated with sonicated (2 min at room temperature) 1 µm sized yellow-green fluorescent carboxylated polystyrene latex beads (Sigma-Aldrich Chemie GmbH) in complete RPMI-1640 culture medium supplemented with 5% FCS (bead:cell ratio of 50:1) for 1 h at 37 ºC. To arrest phagocytosis, the cells were placed on ice for 5–10 min. Following this step, cells were washed with ice cold PBS and incubated with biotin-conjugated anti-CD11b (BD Biosciences, Mountain View, CA, USA) antibody for 30 min at 4 ºC and then again washed with FACS buffer. In the next step, cells were incubated with PerCP-conjugated streptavidin (BD Biosciences) as the second step reagent for another 30 min, washed with FACS buffer and acquired for analysis using a FACSCalibur flow cytometer. Cells incubated with the latex beads at 4 ºC were used to set up the positive/negative cut-off for Latex + cells in FCA. Results were expressed as % of Latex + cells within the CD11b + cell population.

### Reverse transcription-quantitative polymerase chain reaction

Total RNA was extracted by the ABI Prism 6100 Nucleic Acid PrepStation system (Applied Biosystems, Foster City, CA, USA) using the total RNA Chemistry Starter Kit (Applied Biosystems) and DNAse wash solution (Absolute RNA Wash Solution; Applied Biosystems). cDNA was synthesized using the High-Capacity cDNA Reverse Transcription Kit (Applied Biosystems). Triplicate 25-μL RT-qPCR reactions were performed using the TaqMan Gene Expression Master Mix (Applied Biosystems) and premade TaqMan Gene Expression Assays (Applied Biosystems) under the default Applied Biosystems 7500 Real-Time PCR System conditions. All the procedures were described in detail^52^. The following TaqMan Gene Expression Assays were used: CCL19 (Ccl19, Rn01439563_m1), CCL21 (Ccl21, Rn01764651_g1), and β-actin (Actb, Rn00667869_m1). Target mRNA expression was determined using the comparative threshold cycle (dCt) method with β-actin as a reference and SDS v1.4.0. software (Applied Biosystems). Relative amounts of target mRNAs were shown as 2^–dCt^ values, representing the ratio of target to reference genes, where dCt = Ct target – Ct reference.

### Anti-CII antibody ELISA

Serum samples were obtained by centrifugation of blood at 2000*g* for 15 min at 4 °C. Aliquots of sera were de-complemented (56 °C, 30 min) and stored at -20 °C until analysis. The serum level of CII-specific antibodies was assayed by ELISA in 96-well plates (MaxiSorp, Nunc). The plates were coated (50 μL/well) with 5 μg/mL of CII in 50 mM carbonate buffer (pH 9.6) by overnight adsorption at + 4 °C and then incubated with 2% bovine serum albumin (BSA) in PBS (100 μL/well) for 1 h at room temperature. Serum samples were added to the plate (50 μL/well) in duplicate and incubated overnight at + 4 °C. The biotin-conjugated anti-IgG antibody was added to the plate (50 μL/well) and incubated 1 h at room temperature followed by 1 h incubation with a streptavidin–horseradish peroxidase. At each step, the plate was washed with 0.05% Tween 20/PBS (4 × 200 μL/well). Antigen–antibody interactions were visualized using the extrAvidin-peroxidase/o-phenylenediamine system (Sigma, Steinheim, Germany). Dilutions of sera (1: 100), biotin-conjugated anti-IgG antibody (1: 1000; Biolegend Inc., San Diego, CA, USA) and extrAvidin-peroxidase (1: 3000) were prepared in 2% BSA/PBS. The reaction was stopped by the addition of 1 M H_2_SO_4_ (50 μL/well) and absorbance was read at 492/620 nm (A_492/620_). The cutoff value was defined according to the A_492/620_ value obtained from “negative control” wells (2% BSA/PBS) plus 3 × Standard Deviation. Samples were considered positive when the A_492/620_ value exceeded the cut off value.

### Paw tissue culture

Inflamed paws collected by incising at the fur line. Paws from CIA rats were weighed, carefully cut into small pieces and cultured in RPMI 1640 medium (Sigma-Aldrich Chemie GmbH) supplemented with 2 mM l-glutamine (Serva, Heidelberg, Germany), 1 mM Na pyruvate (Serva), 100 U/mL penicillin (ICN, Costa Mesa. CA, USA), 100 μg/mL streptomycin (ICN) and 10% fetal bovine serum, at 37 °C, in a humidified air atmosphere of 5% v/v CO2, for 4 h (for PGE_2_ assay) or overnight (for analyses of NO and cytokine production levels, and redox status parameters), as previously described^28,29,53^. Following extirpation of carrageenan-inflamed paws, soft paw tissues were carefully removed, weighed, cut into small pieces and cultured as described above.

### PGE2, cytokine and NO production

Paw tissue culture supernatants were examined for inflammatory/pain mediators using the following commercial ELISA kits: IL-17A (BioLegend, San Diego, CA, USA), IL-1β (Thermo Scientific, Pierce Biotechnology, Rockford, IL, USA), PGE2, IL-10 and TGF-β (R&D Systems, Minneapolis, MN, USA), according to the manufacturer's instructions. Standard curve was calculated for each assay with limits of detection for IL-17 = 8 pg/ml, IL-1β = 6.5 pg/mL, TNF-α = 2 pg/mL, IL-10 < 10 pg/mL, TGF-β = 4.6 pg/mL and PGE2 < 39 pg/mL. Cytokines and PGE2 concentrations were normalized to paw weight.

The concentration of nitrite, as the end-product of NO production, was measured in the paw tissue culture supernatants using a method based on the Griess reaction^54^. The nitrite concentration was calculated using a NaNO_2_ standard curve with a range from 1 to 40 μM and normalized to paw weight.

### Assessment of redox status parameters

The parameters of redox status were measured in sera and inflamed paw cultures. The levels of superoxide anion radical (O_2_^•^ ‾) were estimated from the rate of reduction of nitroblue tetrazolium (NBT) as described by Auclair and Voisin^55^. Results were expressed as μM of reduced NBT/min/L.

Total oxidant capacity (TOC) was determined by a modified spectrophotometric method using o-dianisidinine^56,57^. The assay was calibrated with hydrogen peroxide (aqueous solution, concentration range 10–200 mmol/L) and the results were expressed in µmol/L hydrogen peroxide per liter (mmol H_2_O_2_ eq./L).

Cu/Zn superoxide dismutase (SOD) activity was determined by a modified spectrophotometric method based on the ability of the SOD enzyme to inhibit the autoxidation of epinephrine in an alkaline medium or bicarbonate buffer 0.05 mmol/L pH 10.2^58^. The SOD activity was calculated as the percentage inhibition of epinephrine autooxidation.

The level of SH-groups (SHG) was determined by Ellman’s method^59^ using 10 mM DTNB (dinitrodithiobenzoic acid) as a reagent. DTNB reacts with aliphatic thiol compounds in the base medium (pH 9.0) and this reaction generates 1 mol of p-nitrophenol anion per mole of thiol. Calibration of the method was achieved using reduced glutathione in the concentration range from 0.1 to 1.0 mM.

Pro-oxidative-antioxidant balance (PAB) was determined by a modified PAB assay using 0.6% 3.3 ', 5,5′-tetramethylbenzidine (TMB) in dimethyl sulfoxide (DMSO) as a chromogen^60^. The PAB test measures the concentration of H_2_O_2_ in an antioxidant environment, as TMB can react at the same time with H_2_O_2_ (a peroxidase-catalyzed reaction) as well as with reducing substances such as uric acid (chemical, non-catalyzed reaction). The enzymatic reaction leads to the oxidation of TMB to a blue product and its reduction to a non-colored product. The net reaction is the difference between the two opposite oxidative and reductive processes on the same substrate. The reaction is calibrated with a mixture of H_2_O_2_ and uric acid at different ratios, ranging from 0 to 100%.

All analyses were performed on ILAB 300 Plus analyzer (Instrumentation Laboratory, Milan, Italy) except the PAB, which was measured on the Spectro star Nano ELISA reader (BMG Labtech, Ortenberg, Germany).

### Statistical analysis

All statistical analyses were performed using GraphPad Prism Version 7 (GraphPad Software, Inc., La Jolla, CA, USA) and SigmaPlot 11 (Systat Software Inc., Richmond, CA) softwares. Data are expressed as mean ± SEM. Differences between groups were tested by Student's unpaired t-test, except differences in paw volume, temperature, and hyperalgesia in carrageenan-induced paw inflammation that were analysed by two-way repeated measures ANOVA followed by Bonferroni test for post hoc comparisons. Values of p ≤ 0.05 were considered significant.

## Results

### Modulatory effects of IR fibres on the autoimmune response, inflammation and burrowing behaviour of CIA-affected rats

#### CIA-affected rats from cages with IR fibre bedding exhibit better burrowing performance compared with their counterparts from cages with standard bedding

**Figure 1:**
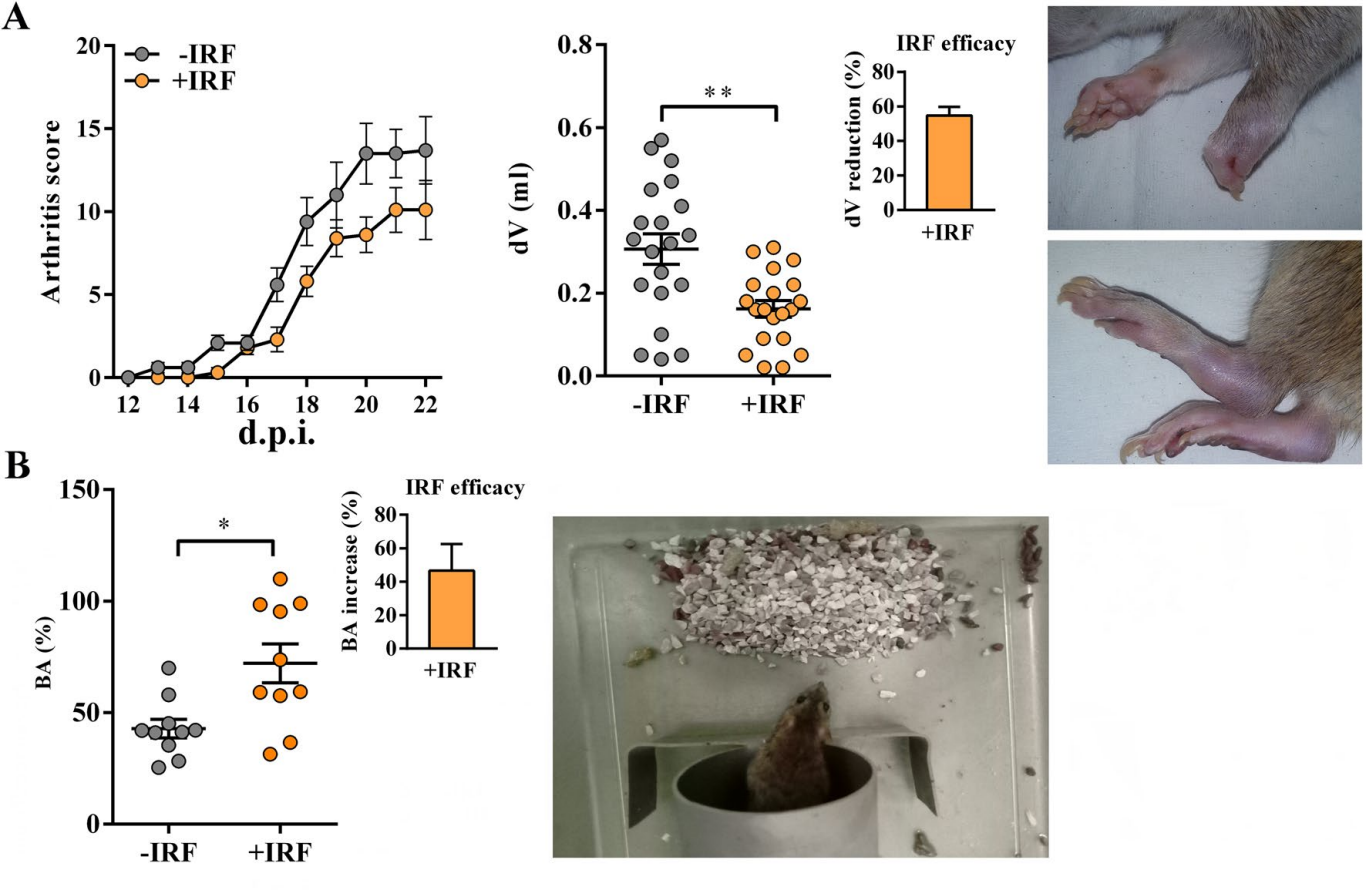
Influence of exposure to IR fibres on daily arthritis score, paw edema and burrowing behaviour in CIA-affected rats. **(A)** Line graph shows daily arthritis score from the 12th–22nd day post-immunisation (d.p.i.) in CIA rats housed in cages with IR-fibre bedding (+IRF rats) or in cages with standard wooden shaving bedding (−IRF rats). Of note, rats were transferred to cages with IR fibre bedding five days before immunization. Scatter plot indicates CIA-induced increase in hind paw volume (dV) in +IRF and −IRF rats. Bar graph shows the dV reduction (%) in +IRF rats relative to −IRF rats (see Material and Methods). Photographs show representative arthritic front and hind paw joints from rat with CIA. (B) Scatter plot indicates the burrowing activity (BA) of each +IRF and −IRF rat expressed as the percentage of the BA before immunisation (basal activity). Bar graph shows the increase in BA (%) in +IRF rats relative to −IRF rats (see Material and Methods). All graphs were created using GraphPad Prism version 7.00 for Windows, GraphPad Software, La Jolla, California, USA (https://www.graphpad.com). Photograph shows a cage-burrow setup and burrowed gravel. Results are expressed as mean ± SEM. n = 10 rats/group. * p ≤ 0.05 and ** p ≤ 0.01.

Generally, the kinetics of CIA development in rats housed in cages with standard bedding followed that described in our previous studies^27,28^ (Fig. S2). In rats housed in cages with IR fibre bedding, the onset of the disease was slightly postponed (from the 13th to the 15th d.p.i. compared with −IRF controls (Fig. 1A). The analysis of the differences in the daily arthritic score using repeated measures two-way ANOVA showed a clear tendency to significance (p = 0.051) in the reduction of the clinical severity of the disease in rats exposed to IR fibres compared with their −IRF counterparts (Fig. 1A). Additionally, the cumulative score of the disease (the sum of daily clinical scores of each individual rat during the observation period) was lower (p ≤ 0.05) in +IRF rats (72.1 ± 9.70) when compared with −IRF ones (47.4 ± 5.56). Rats were also examined for the volumes of inflamed hind paws. In all CIA-affected rats the volumes of hind paws were increased (Fig. 1A). This increase was less (p ≤ 0.01) pronounced in +IRF rats compared with their −IRF counterparts, indicating that the exposure to infrared radiation from IR fibre bedding efficiently reduces the inflammation-induced increase in paw volume (Fig. 1A).

Furthermore, rats were examined for burrowing behaviour. Rats housed in cages with IRF bedding showed better (p ≤ 0.05) burrowing performance compared with −IRF rats (Fig. 1B, Fig. S3).

#### Lower serum levels of serum CII-specific IgG antibodies in CIA-affected rats from cages with IR fibre bedding and diminished IL-17 production in cultures of their hind paws

**Figure 2:**
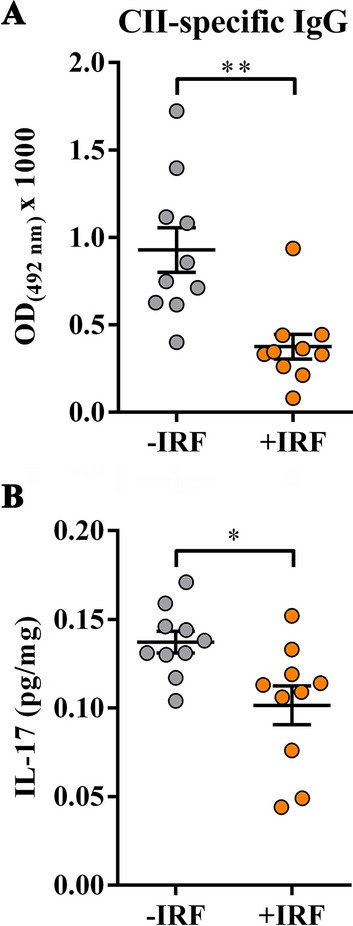
Influence of exposure to IR fibres on the level of CII-specific IgG antibodies in sera and the production of IL-17 in inflamed paws from CIA-affected rats. **(A)** Scatter plot indicates the level of anti-CII IgG antibodies (OD_492nm_ × 1000) in sera from CIA rats housed in cages with IR fibres bedding (+IRF rats) and with standard wooden shaving bedding (−IRF rats). Of note, rats were transferred to cages with IR fibre bedding five days before immunization. **(B)** Scatter plot shows IL-17 production level in the supernatants from hind paw tissue cultures (normalized to the paw weight) from +IRF and −IRF rats. Scatter plots were created using GraphPad Prism version 7.00 for Windows, GraphPad Software, La Jolla, California,USA (https://www.graphpad.com). Results are expressed as mean ± SEM. n = 10 rats/group. * p ≤ 0.05 and ** p ≤ 0.01.

Given that (i) arthritogenic anti-CII antibodies are essential for the development of CIA^61,62^ and (ii) particularly that the arthritogenic antibodies (including those specific for CII) are shown to cause pain even in the absence of overt signs of inflammation^38,63^, serum levels of CII-specific IgG antibodies in CIA rats were examined. Their levels were lower (p ≤ 0.01) in sera from rats transferred to cages with IR fibres bedding (five days before CIPI induction) compared with their counterparts housed into cages with standard bedding (Fig. 2A).

Considering the crucial role of Th17 cells in driving joint damage and consecutive inflammation^64^, the production levels of IL-17, Th17 cell signature cytokine, were examined in supernatants of inflamed paw tissue cultures. The production levels of this cytokine were lower (p ≤ 0.05) in supernatants of inflamed paw tissue cultures from +IRF rats compared with −IRF ones (Fig. 2B).

#### Impaired migration, but greater phagocytic capacity of innate immune CD11b + cells from CIA-affected rats exposed to IR fibres

Considering the role of innate immune CD11b + cells in the development of joint impairment and inflammation in CIA^65^, and the significance of their infiltration into inflamed joint tissue, migratory, adherence and phagocytic capacity of CD11b + cells from CIA rats housed in cages with IR fibre and standard bedding were examined.

**Figure 3:**
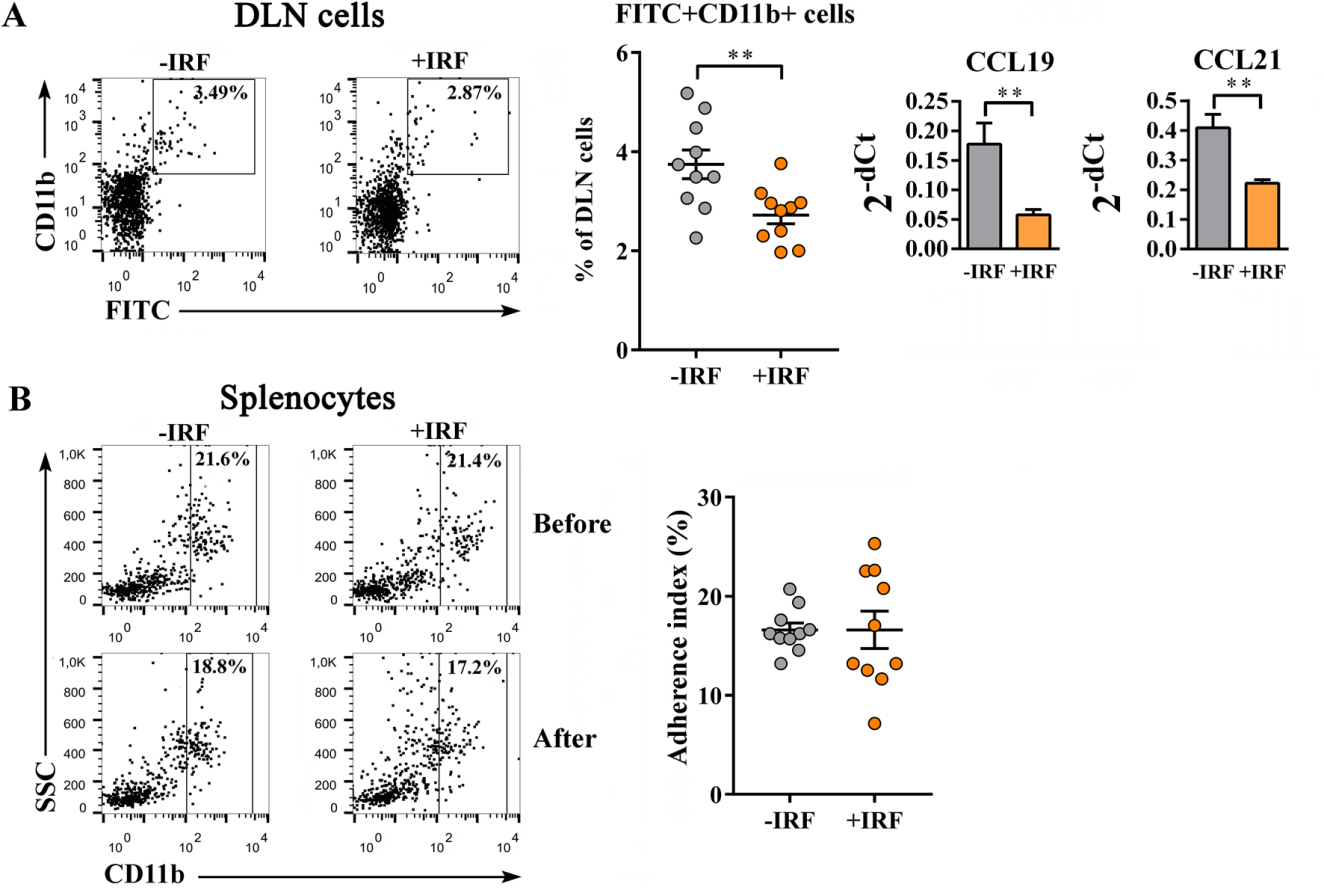
Influence of exposure to IR fibres on the migration and adherence capacity of innate immune CD11b + cells from CIA-affected rats. **(A)** Representative flow cytometry dot plots show the frequency of FITC-stained CD11b + cells in lymph nodes draining the site of FITC application (DLN) in FITC painting test (see Material and Methods) in CIA rats housed in cages with IR fibre bedding (+IRF rats) and with wooden shaving bedding (−IRF rats). Of note, rats were transferred to cages with IR fibre bedding five days before immunization. Bar graphs show the expression of mRNA for CCL19 and CCL21 in DLNs from +IRF and −IRF rats, as determined by RT-qPCR. Results are represented as 2^−dCt^ relative to β-actin. (B) Representative flow cytometry dot plots show CD11b expression on splenocytes from +IRF and −IRF rats before (upper dot plots) and after (lower dot plots) passage through nylon wool in the adherence assay (see Material and Methods). Scatter plot indicates the adherence index, i.e. the percentage of adherent cells of CD11b + cells from spleens of +IRF and −IRF rats (calculated as indicated in Material and Methods). (A, B) Flow cytometry profiles were generated using FlowJo Software for Windows, Version 7.8. FlowJo, LLC, Ashland, Oregon, USA (https://www.flowjo.com). Bar graphs (A) and (B) scatter plot were created using GraphPad Prism version 7.00 for Windows, GraphPad Software, La Jolla California, USA (https://www.graphpad.com). Results are expressed as mean ± SEM. n = 10 rats/group. ** p ≤ 0.01.

The analysis of the frequency of FITC-labelled CD11b + cells in DLNs in the FITC painting test revealed that their frequency was lower (p ≤ 0.01) in +IRF rats than in −IRF controls, indicating impaired migration capacity of CD11b + cells in rats from cages with IR fibre bedding (Fig. 3A). Considering that CD11b cells express CCR7 receptor so that their trafficking is substantially influenced by CCL19 and CCL21 chemokines^50,66^, the expression of mRNAs for these chemokines in DLNs was examined, as well. Indeed, the exposure to IR cage bedding reduced (p ≤ 0.01) the expression of mRNAs for both chemokines (Fig. 3A).

**Figure 4:**
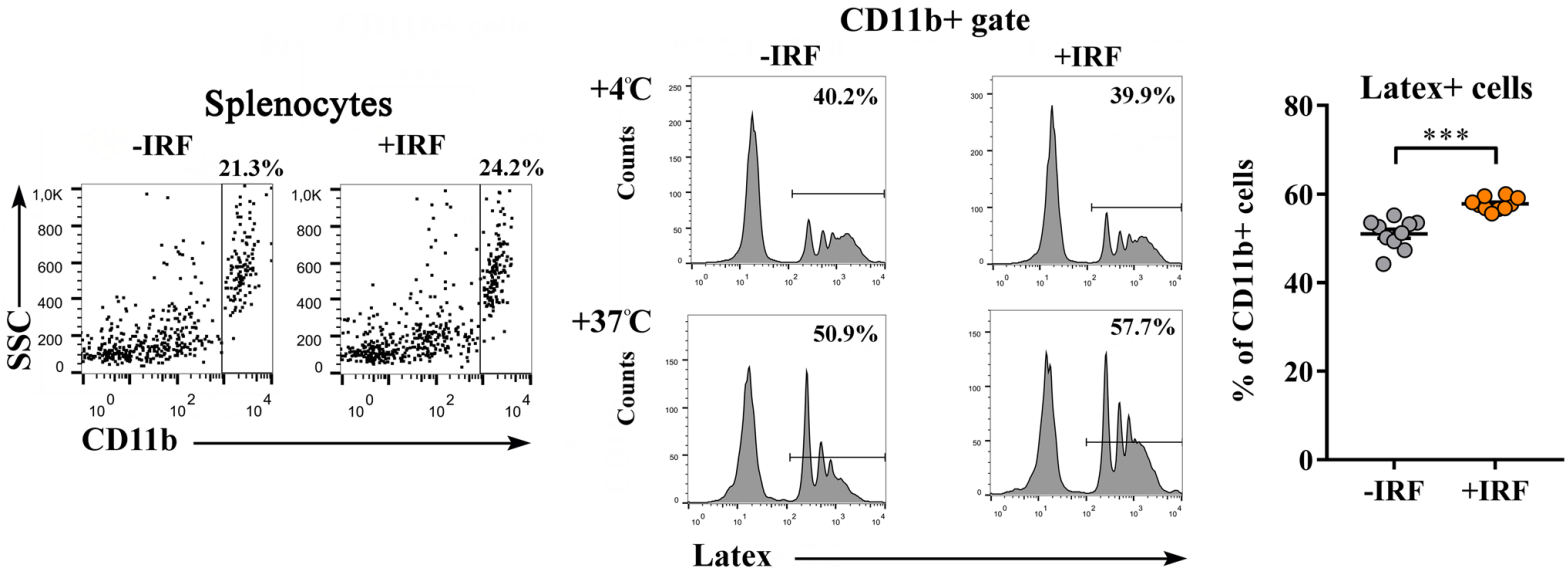
Influence of exposure to IR fibres on the phagocytic capacity of innate immune CD11b + cells from CIA-affected rats. Representative flow cytometry histograms show latex particle phagocytosis at + 4° C and + 37° C by CD11b + cells (gated as shown in representative flow cytometry dot plots generated using FlowJo Software for Windows, Version 7.8. FlowJo, LLC, Ashland, Oregon, USA; https://www.flowjo.com.) from CIA rats housed in cages with IR fibre bedding (+IRF rats) and with standard wooden shaving bedding ( –IRF rats). Rats were transferred to cages with IR fibre bedding five days before immunization. Note that a significant difference between the groups was apparent only at 37° C. Scatter plot (created using GraphPad Prism version 7.00 for Windows, GraphPad Software, La Jolla, California, USA; https://www.graphpad.com) indicates the percentage of phagocyting (latex +) cells among CD11b + cells from the spleen of +IRF and –IRF rats (see Material and Methods). Results are expressed as mean ± SEM. n = 10 rats/group. *** p ≤ 0.001.

Next, considered that in the response to tissue inflammation CD11b + cells leave the spleen en masse to accumulate in injured tissue, and participate in the control of inflammation by phagocyting dead cells and necrotic tissue, and secreting anti-inflammatory/immunoregulatory mediators^67,68^, the adherence and phagocytic capacity of CD11b + cells from spleen were examined. We failed to show any statistically significant difference between the adherence capacity of CD11b + splenocytes from +IRF rats and those from −IRF rats (Fig. 3B). The analysis of Latex bead phagocytosis showed higher (p ≤ 0.001) phagocytic capacity of CD11b + splenocytes from rats exposed to IR fibres had compared with those from rats housed in cages with standard bedding (Fig. 4).

#### Exposure of CIA-affected rats to IR fibres decreases the production levels of pro-inflammatory mediators, but increases production levels of anti-inflammatory/immunoregulatory mediators in cultures of inflamed paws

To elucidate molecular mechanisms underlying less prominent paw swelling and better burrowing performance of +IRF rats, the production of the key pro-inflammatory mediators with algogenic properties (PGE_2_, NO, TNF-α and IL-1β)^69,70^ and anti-inflammatory/immunoregulatory mediators with analgesic properties (IL-10 and TGF-β)^71,72^ was examined in cultures of their hind paws.

**Figure 5:**
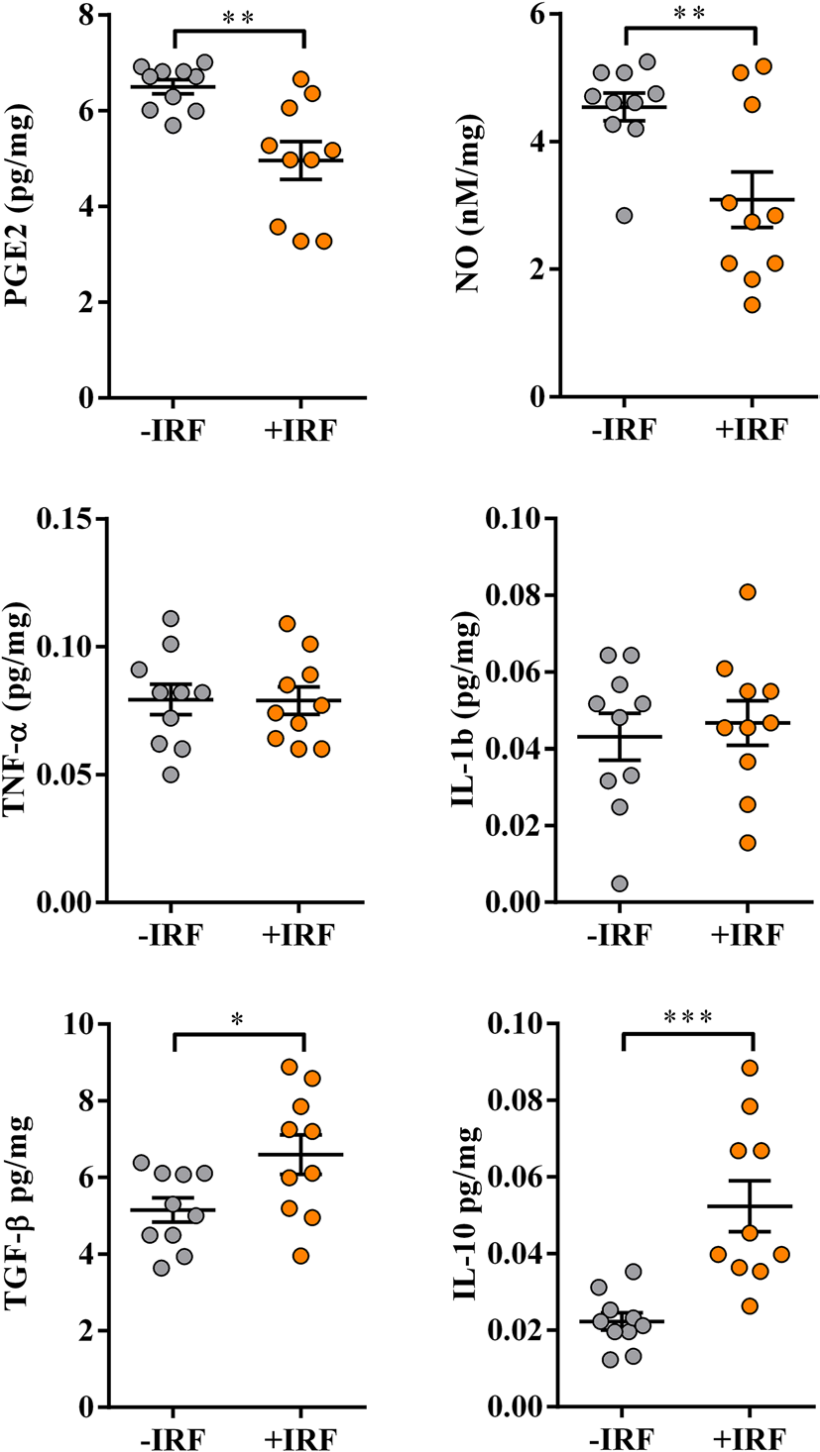
Influence of exposure to IR fibres on the production of PGE_2_, NO and cytokines by inflamed paws from CIA-affected rats. Scatter plots (created using GraphPad Prism version 7.00 for Windows, GraphPad Software, La Jolla, California, USA; https://www.graphpad.com) show PGE_2_, NO, TNF-α, IL-1β, TGF-β and IL-10 production levels in the supernatants from 4 h (PGE2) and overnight hind paw tissue cultures (normalized to the paw weight) (see “Material and methods”) from CIA rats were housed in cages with IR fibre bedding (+IRF) and their counterparts housed in cages with standard wooden shaving bedding (−IRF rats). Rats were transferred to cages with IR fibre bedding five days before immunization. Hind paws were excised from CIA rats on the 22nd day post immunisation. Results are expressed as mean ± SEM. n = 10 rats/group. * p ≤ 0.05, ** p ≤ 0.01 and *** p ≤ 0.001.

The production levels of PGE_2,_ a pro-inflammatory lipid mediator present at high levels in the synovial fluid of patients suffering from RA^73–75^, were lower (p ≤ 0.01) in paw cultures from +IRF rats when compared with those from −IRF rats (Fig. 5).

Additionally, production levels of NO, the pro-inflammatory mediator with an important role in the development of joint injury and inflammation^76^, were also lower (p ≤ 0.01) in inflamed paw cultures from rats exposed to IR-emitting fibres than in those from rats housed in cages with standard bedding (Fig. 5).

On the other hand, we failed to show statistically significant differences in the production levels of pro-inflammatory cytokines (TNF-α and IL-1β), which are shown to significantly contribute to the joint injury and inflammation, and consequently arthralgia in RA^77^ in inflamed paw tissue cultures from +IRF rats and their −IRF counterparts (Fig. 5).

Considering that the ratio between pro-inflammatory to anti-inflammatory/immunoregulatory mediators (IL-10 and TGF-β) in inflamed tissue is more important for RA severity than their absolute levels^37^ and that both IL-10 and TGF-β act as endogenous analgesics^71,72^, their production levels in hind paw cultures were explored, as well. The levels of IL-10 and TGF-β were higher (p ≤ 0.05 and p ≤ 0.001, respectively) in cultures of inflamed paws from +IRF rats compared with IRF rats (Fig. 5).

#### CIA-affected rats from cages with IR fibres exhibit improved serum redox status compared with their counterparts from cages with standard bedding

Given that oxidative stress has been also implicated in the development of inflammation in chronic autoimmune diseases, including RA and its experimental models^78^, the redox status was examined in both sera and inflamed joint tissue cultures.

**Figure 6:**
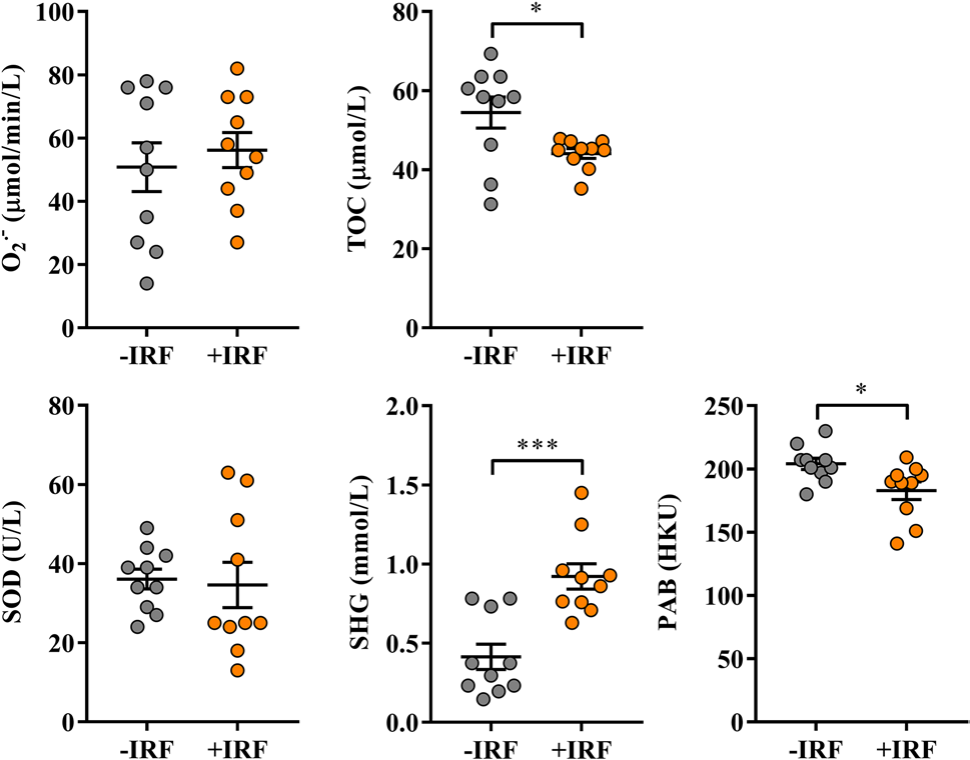
Influence of exposure to IR fibres on the redox status in sera from CIA-affected rats. Scatter plots show pro-oxidant parameters: superoxide anion radical (O2• ^−^) level and total oxidant capacity (TOC); antioxidant parameters: superoxide dismutase (SOD) activity, and sulfhydryl groups (SHG) level; and pro-oxidant-antioxidant balance (PAB) in sera from CIA rats housed in cages with IR fibre bedding (+IRF) and their counterparts housed in cages with standard wooden shaving bedding (−IRF rats). Of note, rats were transferred to cages with IR fibre bedding five days before immunization. Sera were obtained from CIA rats on the 22^nd^ day post immunisation. All graphs were created using GraphPad Prism version 7.00 for Windows, GraphPad Software, La Jolla, California, USA (https://www.graphpad.com). Results are expressed as mean ± SEM. n = 10 rats/group. * p ≤ 0.05 and *** p ≤ 0.001.

The exposure of rats to IR-emitting fibres had no effects on the serum level of O_2_^•^^−^ and SOD activity in CIA rats (Fig. 6). However, lower (p ≤ 0.05) TOC levels accompanied by higher SHG (p ≤ 0.001) levels were found in sera from +IRF rats compared with control rats (Fig. 6). Consistently, lower (p ≤ 0.05) PAB value was detected in sera from +IRF rats compared with −IRF rats (Fig. 6).

We failed to detect any statistically significant differences in the examined redox parameters in the inflamed paw cultures (Fig. S4).

### Housing of rats in cages with IF fibre bedding beginning 5 days before carrageenan administration moderates CIPI development

Given that rats from cages with IR bedding exhibited diminished antibody and Th17 cell responses, and that anti-CII antibodies are found to induce pain behaviour acting directly on sensory neurons, viz. independently of their pro-inflammatory action as pathological antibodies^38^, the study was extended to encompass CIPI model, a “classical” model of the non-autoimmune inflammatory response and inflammatory pain.

#### Exposure to IR fibres reduces temperature, swelling and hyperalgesia of carrageenan-inflamed rat paws and improves burrowing behaviour of CIPI-affected rats

**Figure 7:**
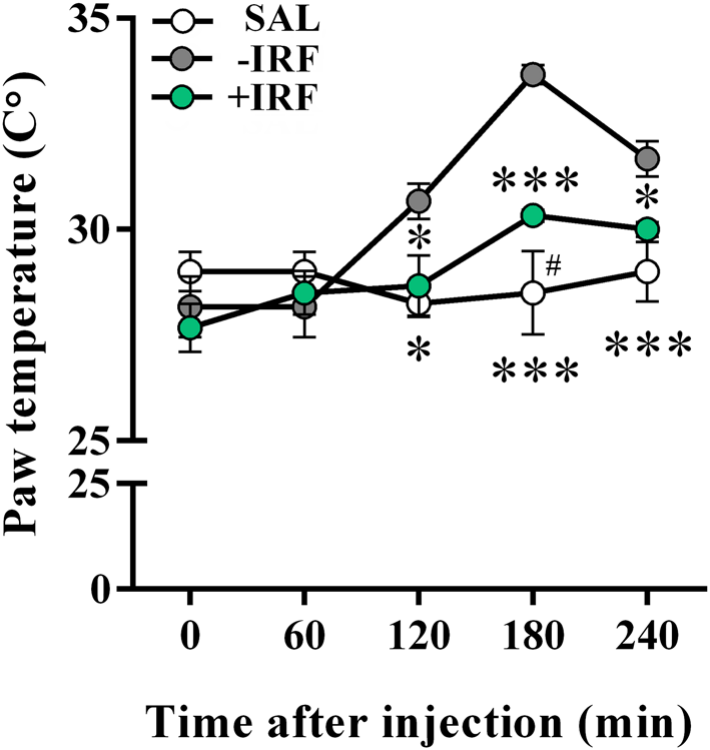
Influence of exposure to IR fibre bedding on the paw temperature of rats with carrageenan-inflamed paws. Rats housed in cages with IR fibre bedding (+IRF) or in cages with standard wooden shaving bedding (−IRF) were injected with carrageenan. Of note, rats were transferred to cages with IR fibre bedding five days before immunization. Rats housed in the cages with standard wooden shaving bedding were injected with saline to serve as an additional control (SAL rats). Line graph (created using GraphPad Prism version 7.00 for Windows, GraphPad Software, La Jolla California USA; https://www.graphpad.com) indicates the surface paw temperature measured before and after carrageenan or saline injection in +IRF, –IRF and SAL rats. Of note, rats were transferred to cages with IR fibre bedding five days before immunization. Results are expressed as mean ± SEM. n = 6 rats/group.* p ≤ 0.05 and *** p ≤ 0.001 vs –IRF; and ^#^ p ≤ 0.05 vs +IRF.

**Figure 8:**
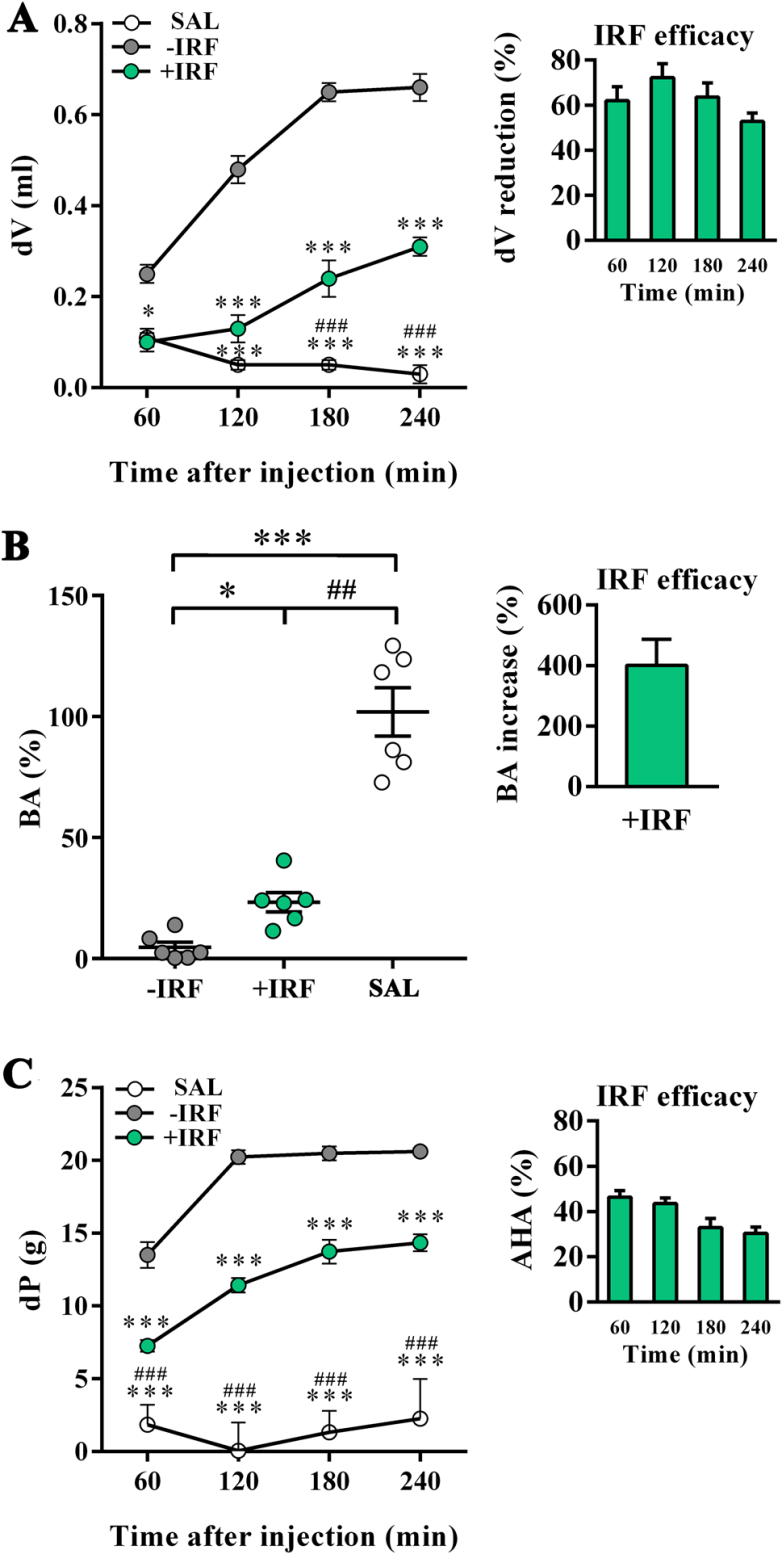
Influence of exposure to IR fibre bedding on paw edema, mechanical stimulus-evoked pain behaviour, and burrowing behaviours of rats with carrageenan-inflamed paws. Right hind paws of rats housed in cages with IR fibre bedding (+IRF) or in cages with standard wooden shaving bedding (−IRF) were injected with carrageenan. Of note, rats were transferred to cages with IR fibre bedding 5 days before immunization. A group of rats with right hind paws injected with saline from cages with standard wooden shaving bedding served as an additional control (SAL rats). **(A)** Line graph indicates the increase in paw volume (dV) in carrageenan or saline administered rats relative to that before the administration. Bar graph shows the dV reduction (%) in +IRF rats relative to −IRF rats (see “Material and methods”). **(B)** Scatter plot indicates the burrowing activity (BA) of each rat following carrageenan or saline administration expressed as the percentage of the BA before the administration (basal activity). Bar graph shows the increase in BA (%) in +IRF rats relative to −IRF rats (see “Material and methods”). **(C)** Line graph indicates the difference between the paw withdrawal thresholds after and before carrageenan or saline injection (dP) in +IRF, –IRF and SAL rats. Bar graph shows the antihyperalgesic activity (AHA) of exposure to IR fibre bedding (+IRF) in rats with carrageenan-induced paw inflammation (see “Material and methods”). All line graphs and scatter plots were created using GraphPad Prism version 7.00 for Windows, GraphPad Software, La Jolla, California, USA (https://www.graphpad.com). Results are expressed as mean ± SEM. n = 6 rats/group.* p ≤ 0.05 and *** p ≤ 0.001 vs −IRF; and ^##^ p ≤ 0.01 ^###^ p ≤ 0.001 vs +IRF.

In rats housed in the cages with IR fibre bedding the surface temperature of carrageenan-injected inflamed paw was lower than in rats housed with standard bedding at 120 min (p ≤ 0.05), 180 min (p ≤ 0.001) and 240 min (p ≤ 0.05) after the injection of carrageenan (Fig. 7). The surface temperature of paws injected with saline did not change during the period of observation, and it was lower when compared with the surface temperature of carrageenan-injected paws from −IRF rats at 120 min (p ≤ 0.05), 180 min (p ≤ 0.001) and 240 min (p ≤ 0.001) post-injection (Fig. 7). Besides, it was lower when compared with the surface temperature of carrageenan-injected paws from +IRF rats at 180 min (p ≤ 0.05) post-injection (Fig. 7). Additionally, in rats housed in the cages with standard bedding, the intraplantar injection of carrageenan elicited paw swelling, as one of the cardinal signs of inflammation (Fig. 8A). In −IRF rats this increase in paw volume was at the maximum at 180 min post injection of carrageenan and remained at this level until the end of observation (Fig. 8A). In the rats transferred to cages with IR fibre bedding five days before the administration of carrageenan, the paw volume gradually increased until 240 min post the injection of carrageenan (Fig. 8A). Control SAL rats exhibited a transient increase in the affected paw volume 60 min after injection of saline, but even at this increase was lower (p ≤ 0.05) than in carrageenan-injected −IRF rats (Fig. 8A). In rats exposed to IRF as cage bedding, the increase in the volume of the carrageenan-administered paw was lower (p ≤ 0.05) at 60 min after the injection than in −IRF rats, and it remained lower (p ≤ 0.001) when examined at later time points (Fig. 8A).

The analysis of burrowing performance showed that carrageenan markedly impaired (p ≤ 0.01) burrowing activity of both −IRF and +IRF rats compared with SAL control rats (Fig. 8B, Fig. S5). However, this carrageenan-induced decrease in burrowing performance was less (p ≤ 0.05) prominent in +IRF rats compared with their −IRF counterparts (Fig. 8B, Fig. S5), indicating that infrared radiation from IRF cage bedding ameliorates burrowing performance of CIPI rats (Fig. 8B, Fig. S5).

Considering that differently from chronic pain conditions, in acute pain models reliability of tests evaluating spontaneous behaviours or activities of rodents in their home environments, as it is burrowing test, have not been systematically evaluated^79^, pain behaviour in CIPI model was also examined using classic mechanical stimulus-evoked von Frey test (Fig. 8C). Using this test we found that, compared with the administration of saline, the administration of carrageenan produced paw hyperalgesia (p ≤ 0.001) at all examined time points (Fig. 8C). Additionally, at all examined time points following carrageenan administration rats housed in cages with IR fibre bedding developed markedly less prominent (p ≤ 0.001) paw hyperalgesia compared with rats housed in cages with standard bedding (Fig. 8C). The analysis of infrared radiation efficacy showed that its anti-hyperalgesic efficacy decreased from 180 min onwards (Fig. 8C).

#### Exposure to IR fibres diminishes production of proinflammatory mediators, but increases production of anti-inflammatory mediators in cultures of carrageenan-inflamed paws

**Figure 9:**
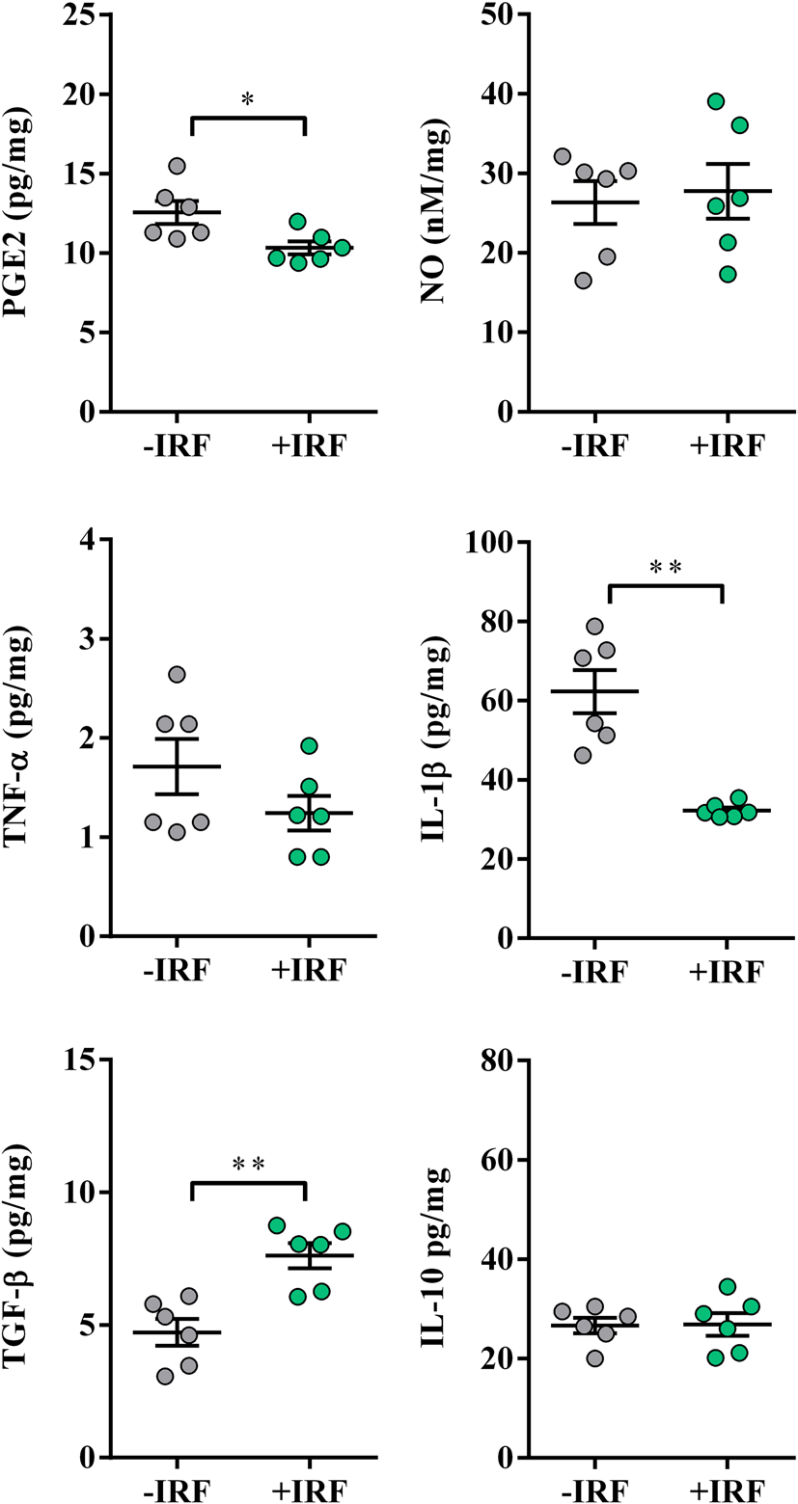
Influence of exposure to IR fibre bedding on the production of PGE2, NO, and cytokines by carrageenan-inflamed paws. Scatter plots (created using GraphPad Prism version 7.00 for Windows, GraphPad Software, La Jolla California USA; https://www.graphpad.com) show PGE2, NO, TNF-α, IL-1β, TGF-β and IL-10 production levels in the supernatants from 4 h (PGE2) or overnight right carrageenan-inflamed hind paw tissue cultures (normalized to the paw weight) (see Material and Methods) from rats housed in cages with IR fibre bedding (+IRF) and with standard wooden shaving bedding (−IRF rats). Results are expressed as mean ± SEM. n = 6 rats/group. * p ≤ 0.05 and ** p ≤ 0.01.

The production of PGE_2_ and NO, pro-inflammatory mediators implicated in the development of CIPI^80^, was examined in the cultures of carrageenan-inflamed paws. The production levels of PGE_2_ were reduced (p ≤ 0.05) in cultures of inflamed paw tissues from +IRF rats compared with their −IRF counterparts (Fig. 9). On the other hand, the production levels of NO were comparable in inflamed paw tissue cultures from +IRF rats and −IRF ones (Fig. 9).

Considering the significant role of TNF-α and IL-1β in development CIPI and related allodynia^81,82^, their production levels in cultures of inflamed paws were also examined. The production of IL-1β by inflamed paw tissues from +IRF rats was lower (p ≤ 0.01) when compared with its production by inflamed paw tissues from −IRF rats (Fig. 9). Differently, the production levels of TNF-α in inflamed paw tissue cultures from +IRF rats did not statistically significantly differ from those in inflamed paw cultures from −IRF rats (Fig. 9).

On the other hand, the production levels of TGF-β were higher (p ≤ 0.01) in cultures of inflamed paw tissues from +IRF rats compared with those from −IRF ones, whereas those of IL-10 were comparable between these cultures (Fig. 9).

### Effects of the exposure of rats affected with CIPI to IRF fibres as cage bedding in the treatment paradigm

#### Exposure to IR fibres in the treatment paradigm reduces swelling and hyperalgesia of carrageenan-inflamed rat paws and improves burrowing behaviour of CIPI-affected rats

**Figure 10:**
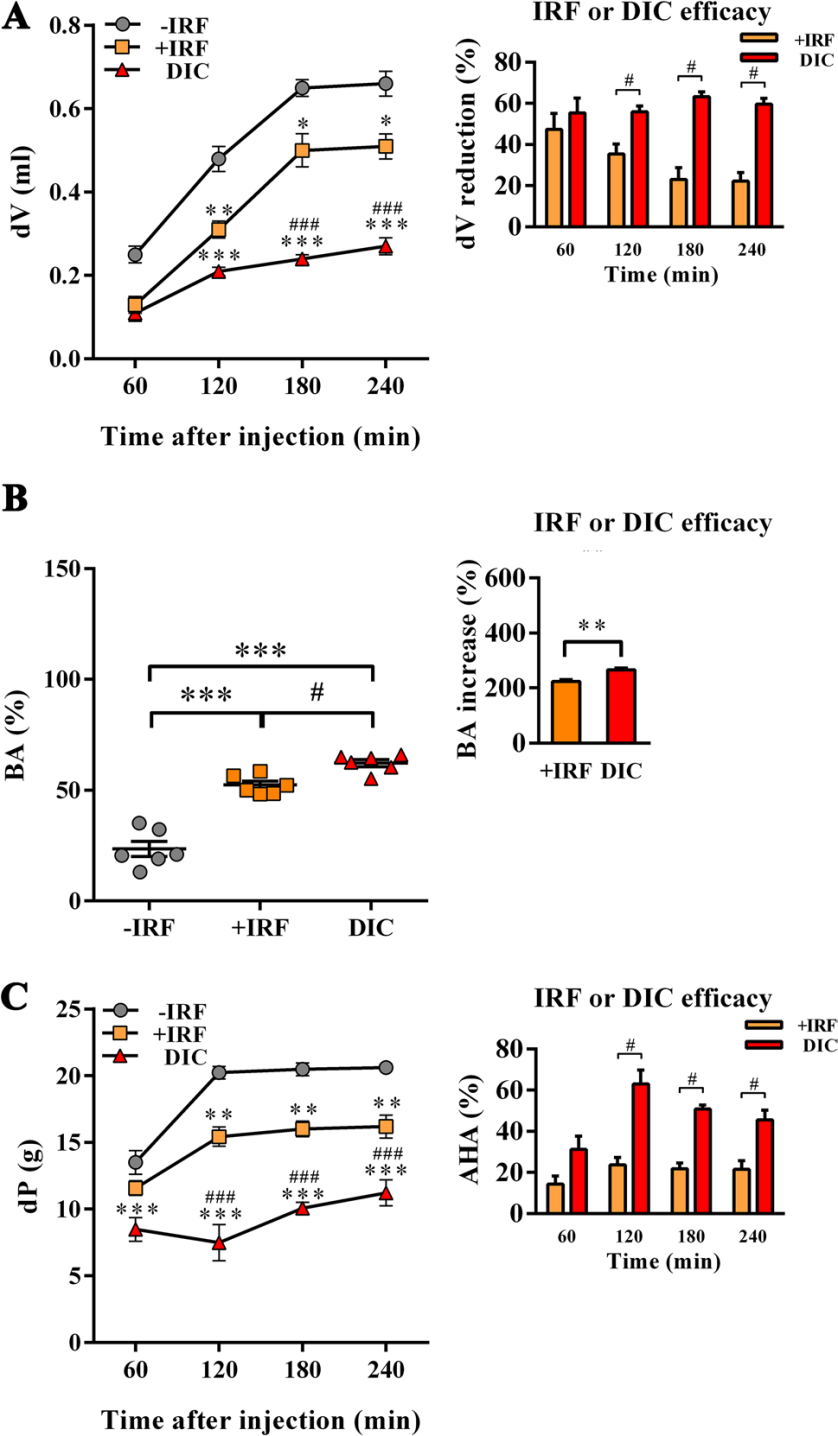
Influence therapeutic exposure to IR fibre on paw edema, mechanical stimulus-evoked pain behaviours and burrowing behaviour of rats with carrageenan-inflamed paws. Immediately after their right hind paws were injected with carrageenan rats were transferred in cages with IR fibre bedding (+IRF rats) or left in standard wooden shaving bedding (−IRF rats). Right paws from a randomly chosen group of −IRF rats were administered with 5 mg/kg Diklofen per os (DIC rats). **(A)** Line graph indicates the increase in paw volume after carrageenan injection (dV) in respect to that before the administration of carrageenan. Bar graph shows the dV reduction (%) in +IRF and DIC rats relative to −IRF rats (see “Material and methods”). (**B)** Scatter plot indicates the burrowing activity (BA) after carrageenan injection in +IRF, −IRF and DIC rats. Bar graph shows the increase in BA (%) in +IRF and DIC rats relative to −IRF rats (see “Material and methods”). **(C)** Line graph indicates the difference between the paw withdrawal thresholds after and before carrageenan injection (dP) in +IRF, –IRF and DIC rats. A bar graph shows the antihyperalgesic activity (AHA) of exposure to IR fibre bedding in a treatment paradigm (+IRF) and Diclofenac treatment (DIC) in rats with carrageenan-induced paw inflammation (see Material and Methods). All line graphs and bar graphs were created using GraphPad Prism version 7.00 for Windows, GraphPad Software, La Jolla, California, USA (https://www.graphpad.com). Results are expressed as mean ± SEM. n = 6 rats/group.* p ≤ 0.05, ** p ≤ 0.01 and *** p ≤ 0.001 vs −IRF; and ^#^ p ≤ 0.05, ^##^ p ≤ 0.01 and ^###^ p ≤ 0.001 vs +IRF.

As described above, the carrageenan-injected paws from rats housed in the cages with standard bedding showed the increase in the volume at the examined time points, but at 180 min following the administration of carrageenan, this increase reached a plateau (Fig. 10A). At 120 min following the administration of carrageenan (p ≤ 0.01) and at all the latter examined time points this increase was less (p ≤ 0.05) prominent in rats transferred to cages with IR bedding following carrageenan administration compared with their counterparts housed in cages with standard bedding (Fig. 10A). Similarly, Diklofen reduced (p ≤ 0.001) the increase in the volume of inflamed paw at all time points from 120 min onwards (Fig. 10A). Comparing the effects of IR fibres and Diklofen on the increase in the volume of inflamed paws, we found that this analgesic drug was more (p ≤ 0.001) efficient than infrared radiation at 120 min (p ≤ 0.001) and 240 min (p ≤ 0.001) following carrageenan administration (Fig. 10A).

The exposure of rats to infrared radiation from cage bedding increased (p ≤ 0.001) their burrowing performance, but to a lower extent (p ≤ 0.05) than the administration of Diklofen (Fig. 10B, Fig. S6). Accordingly, the subsequent analysis of infrared radiation efficacy showed that this treatment was less (p ≤ 0.01) efficient in increasing rat burrowing performance than Diklofen administration (Fig. 10B, Fig. S6).

As expected from the previous experiment, carrageenan increased paw hyperalgesia at all examined time points following carrageenan administration (Fig. 10C). The exposure to IR fibres reduced hyperalgesia (p ≤ 0.01) at 120 min and at all examined time points onward (Fig. 10C). Differently, Diklofen decreased (p ≤ 0.001) hyperalgesia at all examined time points (Fig. 10C). Compared with Diklofen infrared radiation from the cage bedding was less efficient (p ≤ 0.001) in this respect at all examined time points (Fig. 10C). The analysis of Diklofen and infrared fibres anti-hyperalgesic efficacy showed that Diklofen was more efficient at 120 min, 180 min, and 240 min that the exposure to IR fibres, whereas their efficacy was comparable at 60 min (Fig. 10C).

#### Exposure to IR fibres in treatment paradigm diminishes production of proinflammatory mediators, but increases production of anti-inflammatory mediators in cultures of carrageenan-inflamed paws

**Figure 11:**
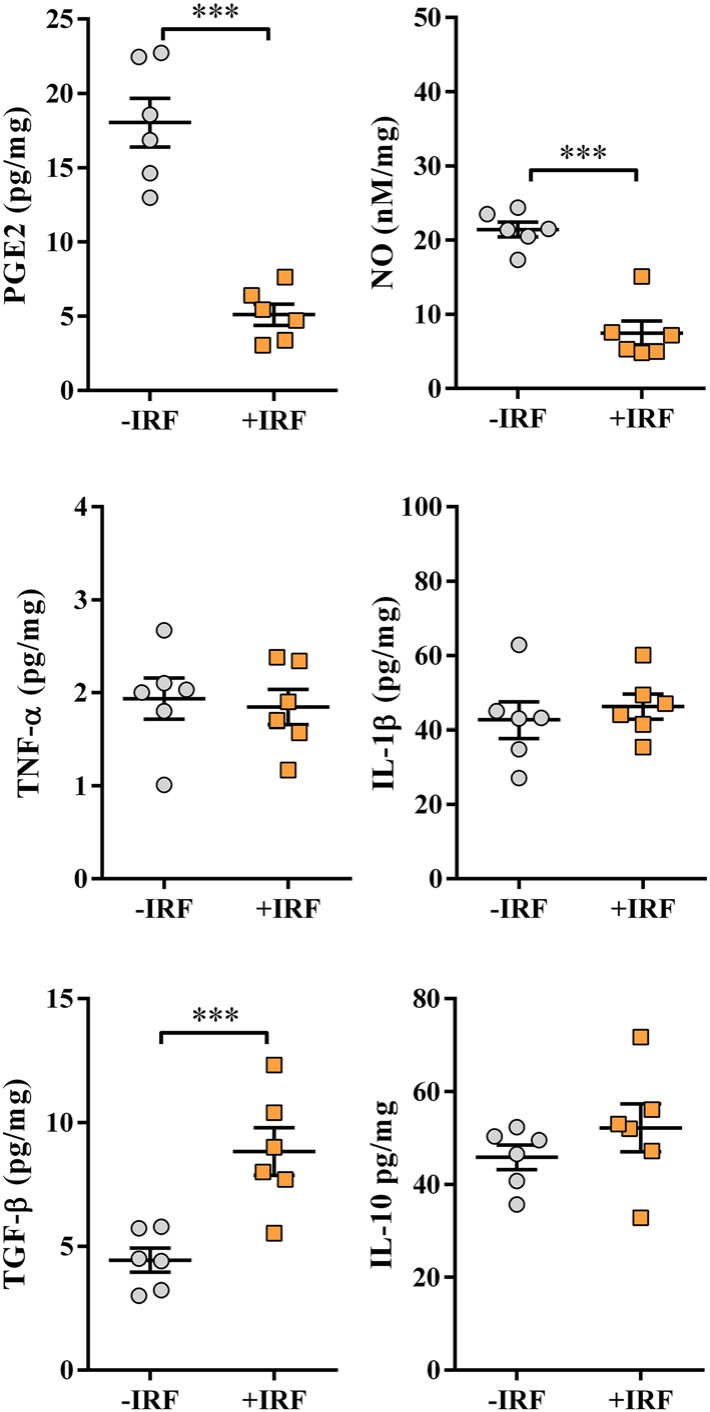
Influence of rat exposure to IR fibre bedding in a treatment paradigm on the production of PGE2, NO, and cytokines by carrageenan-inflamed paws. Scatter plots (created using GraphPad Prism version 7.00 for Windows, GraphPad Software, La Jolla, California, USA; https://www.graphpad.com) show PGE2, NO, TNF-α, IL-1β, TGF-β and IL-10 production levels in the supernatants from 4 h (PGE2) or overnight carrageenan-inflamed paw tissue cultures (normalized to the paw weight) (see Material and Methods) from rats transferred in cages with IR fibre bedding (+IRF rats) or in cages with standard wooden shaving bedding (−IRF rats) immediately following administration of carrageenan. Results are expressed as mean ± SEM. n = 6 rats/group. *** p ≤ 0.001.

In accordance with the effects of IRF on paw inflammation, the production levels of PGE2 and NO were diminished (p ≤ 0.001) in cultures of inflamed paw tissues from rats exposed to IRF as cage bedding in immediacy following e treatment carrageenan administration when compared with those from inflamed paw tissues from rats left in cages with standard bedding (Fig. 11).

The production of proinflammatory cytokines (IL-1β and TNF-β) was comparable in inflamed paw tissue cultures from −IRF and +IRF rats (Fig. 11).

On the other hand, the production levels of TGF-β were higher (p ≤ 0.001) in inflamed paw tissue cultures from +IRF rats compared with −IRF rats, whereas IL-10 production levels were comparable in inflamed paw tissue cultures from these two groups of rats (Fig. 11).

## Discussion

The study showed clearly effects of IR cage bedding constructed from Celliant fibre on the burrowing performance (reflecting a global pain score) in CIA rat model. Generally, mechanisms underlying joint pain in CIA involve direct action of inflammatory mediators and/or CII-specific antibodies on nociceptors, specialized primary afferent sensory neurons in the pain pathway^38,83^. Our results, for the first time to the best of our knowledge, showed that infrared radiation may diminish the generation of (auto)antigen-specific potentially harmful IgG antibodies. By using modified anti-CII antibodies and transgenic or chimeric mice it was established that the interaction of these antibodies with the IgG receptor (FcγRI) on sensory neurons^84^ is important for their pronociceptive properties^38^. These findings are suggested to be of translational value^38^. In favor of the inflammation-independent pronociceptive properties of CII-specific antibodies, are data indicating that: (i) joint pain precedes the appearance of visible signs of arthritis in both RA and CIA, and (ii) depending on the levels of joint tissue-specific antibodies, the perception of pain may differ between individuals with otherwise similar severity of joint inflammation^38^. In keeping with these data, our study showed that rats housed in cages with IR bedding exhibited less prominent burrowing deficit although clinical arthritis score of the disease was only slightly, but not statistically significantly, lower compared with those housed in cages with standard bedding. However, given that CII-specific antibodies form immune complexes that bind to joint cartilage leading to attraction of inflammatory cells (a key step in the development of arthritis)^85^, their contribution to burrowing deficit via promotion of tissue damage and inflammation could not be ruled out. Indeed, our results showed the increase in the average volume of the paws (inflammatory parameter which is not included in the arthritic score) was lower from +IRF rats compared with their −IRF counterparts. This suggested the beneficial effect of infrared radiation on paw swelling. Consequently, the less pronounced compression of edematous fluid on nociceptors may be expected. This finding most likely did not solely reflect the effect of infrared radiation on the degree of CII-specific antibody-mediated tissue injury, but possibly also its influence on the paw infiltration with monocytes/macrophages, as suggested by analysis of CD11b + cell migration in the painting test. To the best of our knowledge, there is no data on the influence of infrared radiation on monocyte/macrophage migration. However, there is data indicating that short exposure to near-infrared laser light may influence the migration of migratory dendritic cells^86^. Thus, it may be speculated that the rat exposure to infrared radiation from IR bedding impaired CD11 + dendritic cell migration into DLN. This could affect follicular helper T cell priming and consequently their capacity to provide help to the cognate germinal center B cells to differentiate into antibody-producing plasma cells^88^. To corroborate this assumption, our results showed that the exposure to IR fibres down-regulated the expression of CCL19 and CCL21, the key chemokines governing not only dendritic cell migration, but also their positioning within DLN, which is crucial for their interactions with T cells and productive T cell response^87,88^. Additionally, in favor of our assumption could be also added that the production of IL-17, Th17 signature cytokine, was diminished in inflamed paw cultures from +IRF rats compared with their −IRF counterparts. Furthermore, considering that the development of autoimmune response-induced damage and consequently inflammation critically depends on the magnitude of the autoimmune response in LN draining the site of immunisation^89^, the delayed appearance of the first signs of inflammation in +IRF rats compared with their –IRF counterparts is also consistent with the negative influence of infrared radiation from IR cage bedding on the development of humoral and cellular autoimmune response in this rat CIA model. To corroborate different effects of the infrared radiation on dendritic cell and monocyte/macrophage migration are data indicating that their migration is influenced by many factors^90^, and that all of them are not equally important for these two types of innate immunity cells^66^.

To further assess mechanisms underlying the development of autoimmune inflammation and inflammatory pain in CIA, the production levels of inflammatory mediators in inflamed paw culture supernatants (mimicking their content in “inflammatory soup”) were examined. The results showed that the infrared radiation-induced changes in the production of inflammatory mediators were the mediator specific. Namely, the decrease in the levels of proinflammatory mediators (PGE_2_ and NO) was followed by the increase in the levels of anti-inflammatory/immunoregulatory mediators (IL-10 and TGF-β), suggesting that infrared radiation did not affect only autoimmune response, but also inflammatory response. Considering the central role of monocytes/macrophages in inflammation in RA/CIA^22^, the shift in the inflammatory mediator profile in supernatants from inflamed CIA rat paws could be related to the enhanced phagocytic capacity of CD11b + cells from +IRF rats compared with their −IRF counterparts. Namely, the phagocytosis of apoptotic cells has been shown to change monocytes/macrophages secretory profile from pro-inflammatory into anti-inflammatory/immunoregulatory profile^91^. Several lines of findings taken conjointly support our results. Firstly, far infrared radiation was shown to augment phagocytosis^92^. Secondly, even short-term exposure of mice affected by chemically induced peritonitis to far infrared radiation may decrease the production of pro-inflammatory mediators by peritoneal macrophages^9^ Thirdly, endothelial cell exposure to far infrared radiation in vitro enhanced the expression of heme oxygenase-1^10^, the enzyme promoting anti-inflammatory response of monocytes/macrophages^93–95^. In keeping with our findings suggesting anti-inflammatory (independent on immunomodulatory) action of infrared radiation from cage bedding are data indicating that far infrared radiation from bioceramics may produce an anti-inflammatory effect on lipopolysaccharide-induced knee joint arthritis in rabbits^96^. Moreover, it should be pointed that the reduced NO production in inflamed tissues from +IRF rats could contribute to the less prominent inflammatory response in +IRF rats through lessening NO-mediated breakdown of cartilage tissue^97,98^. To add additional weight to our finding are data indicating that the exposure to far infrared radiation may negatively affect NO synthesis in inflamed tissue^9^.

To further evaluate the anti-inflammatory properties of infrared radiation, we investigated the development of CIPI in rats transferred to cages with bedding constructed from IR fibres five days before carrageenan administration. We found that paw inflammation (judging by the increase in paw volume, and paw pain) was markedly less prominent in these rats compared with their counterparts from cages with standard bedding. The analysis of inflammatory mediator production in carrageenan-inflamed paw cultures showed that the exposure to infrared radiation led to the shift in inflammatory mediator profile towards a more anti-inflammatory/immunomodulatory phenotype, as the production levels of PGE_2_ and IL-1β decreased, whereas that of TGF-β increased. This finding may indicate that the effects of infrared radiation from IR ceramic particles incorporated into fibres on inflammatory mediator production depend on the type of inflammation and/or duration of the exposure period. It should be underscored that the exposure of rats to IR fibres immediately after carrageenan administration (treatment paradigm) also reduced the paw edema and exerted a beneficial effect on both mechanical-stimulus evoked pain (as shown by von Frey test) and burrowing performance. These effects of infrared radiation were less prominent than those induced by Diklofen. However, given that nonsteroidal anti-inflammatory drugs, including diclofenac, cannot entirely control severe joint pain in RA and that these drugs have (particularly at higher doses) many side-effects, it is highly likely that a combination of the exposure of inflamed joints to IR emitting material and usage of non-steroidal inflammatory drugs could (i) be more efficient in the control of this RA symptom than usage of nonsteroidal anti-inflammatory drugs alone and (ii) allow usage of these drugs at lower doses to achieve the optimal therapeutic effect. According to our literature search, the effects of infrared radiation on inflammation/pain have not been compared in other studies. The analysis of inflammatory mediator production in carrageenan-inflamed paw cultures showed that the exposure to IR fibres in the treatment paradigm also decreased the production of proinflammatory mediators (PGE_2_ and NO), but increased the production of TGF-β. Thus, it may be speculated that effects of infrared radiation from Celliant fibres on inflammatory mediator production depends on the duration of exposure to these fibres, although the influence of the temporal relationship between the treatment initiation and inflammatory stimulus application could not be completely excluded.

Furthermore, it should be pointed out that infrared radiation could influence the pain behaviour of CIA and CIPI rats, not only by reducing the intensity of inflammation and consequently the degree of tissue edema, but also by modulating the secretion of inflammatory mediators, which modulate the peripheral and central nervous sensitization and thereby nociception. The reduced level of PGE_2,_ a classic lipid inflammatory mediator shown to exert algogenic effects by increasing peripheral and central nervous sensitization^83^, in paw cultures from +IRF rats could be associated with inhibitory effects of far infrared radiation from ceramic material on cyclooxygenase-2 expression and PGE_2_ synthesis by cultured macrophages and/or chondrocyte cell lines^99^. It should be pointed out that PGE_2_ may also exert pro-nociceptive effects indirectly, by promoting the differentiation of Th cells producing IL-17^100^. Thus, the reduced production of IL-17 in paw cultures from CIA rats seems to be consistent with the reduced PGE_2_ production. It should be added that IL-17 does not have only an essential role in the development of joint inflammation and thereby in the inflammatory pain in CIA-affected rats^28^, but also participate in the development of joint pain through sensitization of sensory neurons^101^. Additionally, in rats suffering from CIPI, the reduced production of IL-1β may contribute to the moderating effects of IR fibres on inflammatory pain. In favor of this assumption are findings indicating that exposure to infrared radiation from a pad impregnated with IR ceramic placed on the bottom of the housing unit decreases IL-1β production in skin tissue from paws administered with complete Freund’s adjuvant to induce inflammation^102^. Furthermore, beneficial effects of infrared radiation from cage bedding on rat pain behaviour in both CIA and CIPI models may reflect the enhanced production of IL-10 and/or TGF-β in inflamed tissue, as they are shown to moderate peripheral and central sensory nerve sensitization, and thereby nociception^103–106^. To support this notion are data indicating that the exposure to far infrared radiation from ceramic materials diminishes IL-10 production from complete Freund’s adjuvant-inflamed mice paws^102^. In favor of the contribution of diminished production of TGF-β in inflamed tissues to the moderating effect of infrared radiation from cage bedding on inflammatory pain in CIA and CIPI models are literature reports indicating that: (i) TGF-β family members act as modulators of acute and chronic pain perception through the transcriptional regulation of genes encoding endogenous opioids^106^, and (ii) the anti-hyperalgesic effects of IR ceramics involve the opioid system^107^.

Finally, it should be pointed that CIA is associated with adverse changes in the blood redox state reflecting not only joint cartilage destruction, but also the development of inflammatory changes in other tissues or organs^108^. Thus, the increased burrowing capacity, reflecting reduced global pain score and consequently better animal welfare^46^ in +IRF rats compared with their −IRF counterparts was consistent with the improved redox status in their blood, as indicated by PAB. This finding was corroborated by previous studies indicating the beneficial effects of IR ceramics on the systemic levels of oxidative stress biomarkers^109^.

In conclusion, our study (i) showed that the exposure of rats to infrared radiation from IR cage-bedding moderates the development of autoimmune joint inflammation, and consequently has beneficial effects on global spontaneous pain score and welfare and (ii) pointed to rather complex immunomodulatory and anti-inflammatory mechanisms underlying this phenomenon. While further translational studies are warranted, our findings support the use of infrared emitting bed linen, bandages, or garments as adjuvant non-pharmacological treatments for RA patients, and possibly for those suffering from other inflammatory antibody-mediated autoimmune diseases. Additionally, the study showed that the exposure to such infrared radiation may have also beneficial effect on the development of sterile inflammation and the inflammatory pain, as well as a therapeutic effect when a sterile inflammation is induced; the findings that also may have significant translatory value.

## Supplementary Information

Supplementary Figures.
