# Supplementary material for: Infrared radiation from cage bedding moderates rat inflammatory and autoimmune responses in collagen-induced arthritis
Source: Sci Rep. 2021 Feb 3;11:2882. doi: 10.1038/s41598-021-81999-7 (PMC7858598; doi:10.1038/s41598-021-81999-7)
Supplement: Supplementary file 1 — Supplementary Figures. [file 41598_2021_81999_MOESM1_ESM.docx]

**Infrared radiation from cage bedding moderates rat inflammatory and autoimmune responses in collagen-induced arthritis**

*Short title: Celliant bedding moderates autoimmune and inflammatory responses*

Jasmina Djuretić^1#^, Mirjana Dimitrijević^2#^, Marija Stojanović^1^, Jelena Kotur Stevuljević^3^, Michael R Hamblin^4^, Ana Micov^5^, Radica Stepanović-Petrović^5^, Gordana Leposavić^1*^

^#^equally contributed

^1^Department of Pathobiology, Faculty of Pharmacy, University of Belgrade, Vojvode Stepe 450, Belgrade, Serbia

^2^Department of Immunology, Institute for Biological Research "Siniša Stanković" – national Institute of Republic Serbia, University of Belgrade, Bulevar despota Stefana 142, Belgrade, Serbia

^3^Department of Biochemistry, Faculty of Pharmacy, University of Belgrade, Vojvode Stepe 450, Belgrade, Serbia

^4^ Laser Research Centre, Faculty of Health Science, University of Johannesburg, Doornfontein 2028, South Africa

^5^ Department of Pharmacology, Faculty of Pharmacy, University of Belgrade, Vojvode Stepe 450, Belgrade, Serbia

*Correspondence:

Gordana Leposavić, MD PhD

Tel: +381 64 1105075

E-mail: [gordana.leposavic@pharmacy.bg.ac.rs](mailto:gordana.leposavic@pharmacy.bg.ac.rs)

**Supplementary Figure S1.** The photograph shows a cage with Celliant bedding placed over the standard wood shaving, side view (see Material and Methods).

**
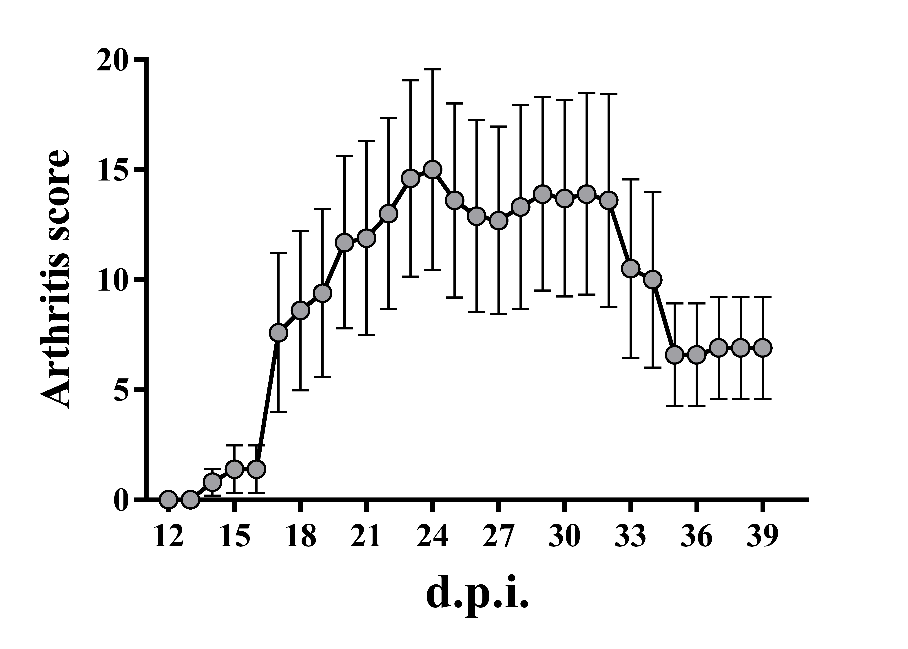
**

**Supplementary Figure S2.** Daily arthritis score (mean ± SEM) in female DA rats (n = 10) from the 12^th^ – 39^th^ day post-immunisation (d.p.i.) with bovine collagen type II in incomplete Freund's adjuvant. Line graph was created using GraphPad Prism version 7.00 for Windows, GraphPad Software, La Jolla California USA (www.graphpad.com).


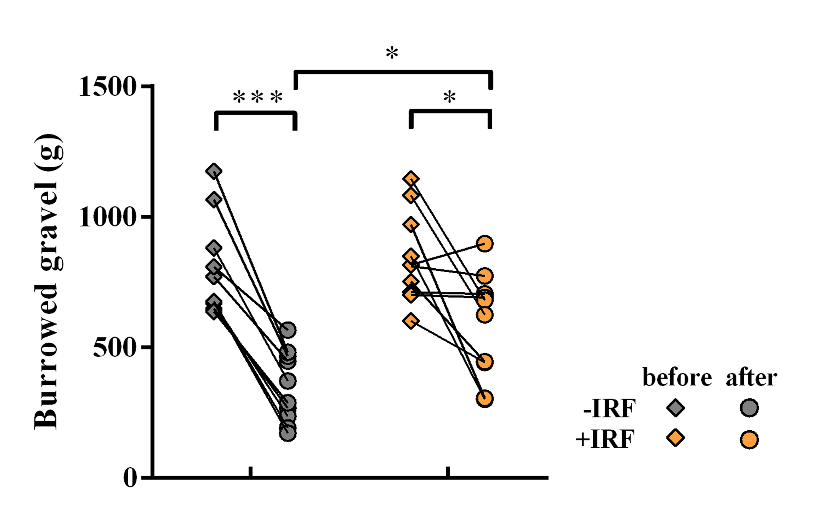


**Supplementary Figure S3. Exposure to IR fibre bedding improved the burrowing behaviour of CIA-affected rats.** Scatter plot (created using GraphPad Prism version 7.00 for Windows, GraphPad Software, La Jolla California USA; www.graphpad.com) indicates the amount of burrowed gravel (g) by CIA rats housed in cages with IR fibre bedding (+IRF rats) and with standard wooden shaving bedding (-IRF rats) in burrowing tests performed before and after immunisation. Of note, rats were transferred in cages with IR fibre bedding beginning from five days before immunisation. n = 10 rats/group. *p ≤ 0.05 and *** p ≤ 0.001.


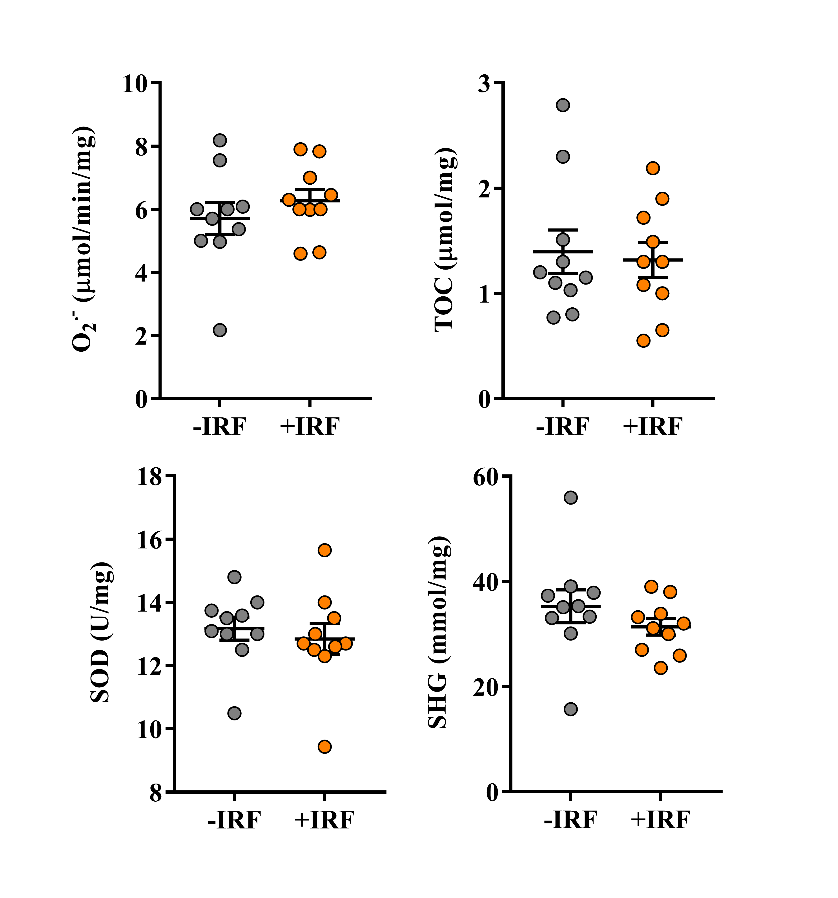


**Supplementary Figure S4. Influence of exposure to IR fibres on the redox status in cultures of hind paw tissues from CIA-affected rats.** Scatter plots (created using GraphPad Prism version 7.00 for Windows, GraphPad Software, La Jolla California USA; www.graphpad.) show pro-oxidant parameters: superoxide anion radical (O2• ‾) level and total oxidant capacity (TOC); antioxidant parameters: superoxide dismutase (SOD) activity and sulfhydryl groups (SHG) level in the supernatants from hind paw tissue cultures (normalized to the paw weight) of CIA rats housed in cages with IR fibre bedding (+ IRF rats) or in and with standard wooden shaving bedding (-IRF rats). Of note, rats were transferred in cages with IR fibre bedding five days before immunization. Results are expressed as mean ± SEM. n = 10 rats/group.


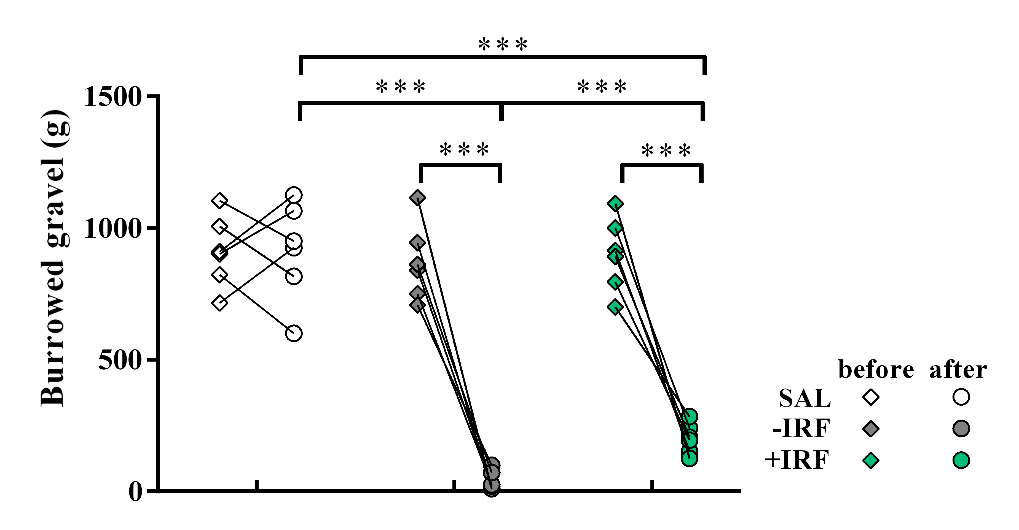


**Supplementary Figure S5. Rats with carrageenan-inflamed paws from cages with IR fibre bedding exhibited better burrowing performance than those from cages with standard bedding.** Right hind paws of rats from cages with IR fibre bedding (+IRF rats) and with standard wooden shaving bedding (-IRF rats) were administered with carrageenan. Of note rats, were transferred to cages with IR fibre bedding five days before immunization. A group of rats from cages with standard wooden shaving with right hind paws injected with saline (SAL rats) to serve as an additional control (SAL rats). Scatter plot (created using GraphPad Prism version 7.00 for Windows, GraphPad Software, La Jolla California USA; www.graphpad.com) indicates the amount of burrowed gravel (g) by +IRF, -IRF and SAL rats in burrowing tests performed before and after carrageenan or saline injection. n = 6 rats/group. *** p ≤ 0.001.

**
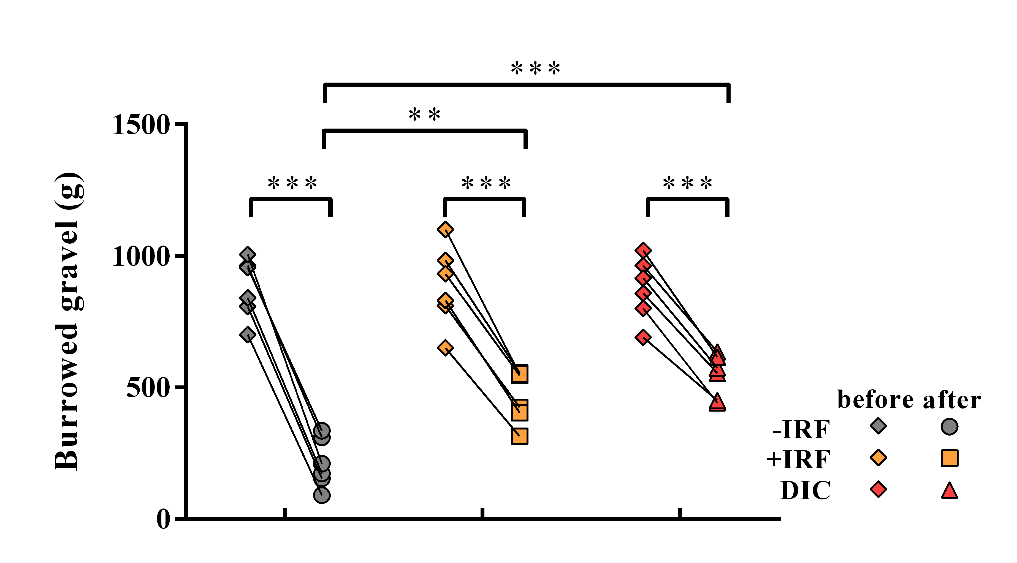
**

**Supplementary Figure S6. Therapeutic exposure of rats with paws injected with carrageenan to IR fibre bedding improved their burrowing behaviour.** Rats with paws injected with carrageenan were immediately placed in cages with IR fibre bedding (+IRF rats) or left in cages with standard wooden shaving bedding (-IRF rats). A randomly chosen group of rats from cages with standard bedding was administered with 5 mg/kg Diklofen per os immediately after carrageenan administration (DIC rats) (see Material and methods). Scatter plot (created using GraphPad Prism version 7.00 for Windows, GraphPad Software, La Jolla California USA; www.graphpad) indicates the amount of burrowed gravel (g) by +IRF, -IRF and DIC rats in burrowing tests performed before and after carrageenan injection. n = 6 rats/group. ** p ≤ 0.01 and *** p ≤ 0.001.
